# Resolving a century-old case of generic mistaken identity: polyphyly of *Chitoniscus* sensu lato resolved with the description of the endemic New Caledonia *Trolicaphyllium* gen. nov. (Phasmatodea, Phylliidae)

**DOI:** 10.3897/zookeys.1055.66796

**Published:** 2021-08-05

**Authors:** Royce T. Cumming, Stéphane Le Tirant, Thies H. Büscher

**Affiliations:** 1 Montreal Insectarium, 4101 rue Sherbrooke est, Montréal, Québec, H1X 2B2, Canada Montreal Insectarium Montréal Canada; 2 Richard Gilder Graduate School, American Museum of Natural History, New York, NY 10024, USA American Museum of Natural History New York United States of America; 3 Biology, Graduate Center, City University of New York, NY, USA City University of New York New York United States of America; 4 Department of Functional Morphology and Biomechanics, Zoological Institute, Kiel University, Am Botanischen Garten 9, 24118, Kiel, Germany Kiel University Kiel Germany

**Keywords:** Camouflage, Drehu, Grande Terre, Ile de Bélep, L’Île-des-Pins, Lifou, Lifu, Maré, mimicry, new combination, Phasmida, Tiga, walking leaf

## Abstract

With every molecular review involving *Chitoniscus* Stål, 1875 sensu lato samples from Fiji and New Caledonia revealing polyphyly, the morphology from these two distinct clades was extensively reviewed. Morphological results agree with all previously published molecular studies and therefore *Trolicaphyllium***gen. nov.** is erected to accommodate the former *Chitoniscus* sensu lato species restricted to New Caledonia, leaving the type species *Chitoniscuslobiventris* (Blanchard, 1853) and all other Fijian species within *Chitoniscus* sensu stricto. Erection of this new genus for the New Caledonian species warrants the following new combinations: *Trolicaphylliumbrachysoma* (Sharp, 1898), **comb. nov.**, *Trolicaphylliumerosus* (Redtenbachher, 1906), **comb. nov.**, and *Trolicaphylliumsarrameaense* (Größer, 2008a), **comb. nov.** Morphological details of the female, male, freshly hatched nymph, and egg are illustrated and discussed alongside the *Chitoniscus* sensu stricto in order to differentiate these two clades which have been mistaken as one for decades.

## Introduction

**Figure 1.**
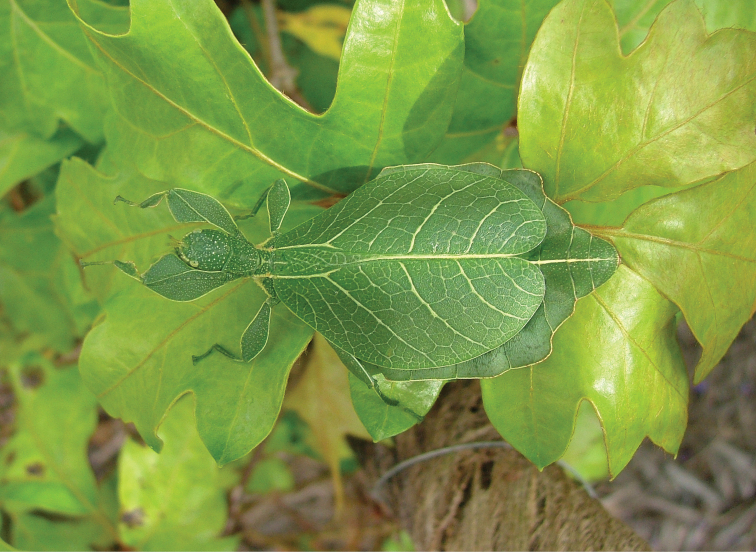
Dorsal habitus of a live adult female *Trolicaphylliumsarrameaense* comb. nov. photographed by Thierry Salesne (New Caledonia) in March 2011, in Vallée Pierrat, La Foa, Grand Terre.

**Figure 2.**
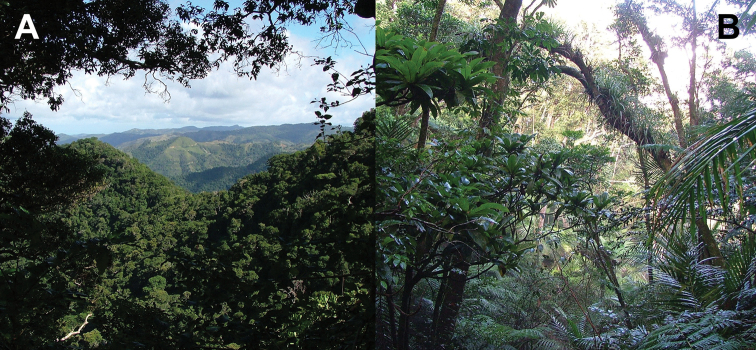
Example of *Trolicaphyllium* gen. nov. habitat on Dogny Plateau, Sarramea Commune, photographed by Thierry Salesne (New Caledonia) in August 2011, at approximately 1,000 meters elevation **A** view from the trail over the plateau **B** view of the primary forest undergrowth.

Phasmatodea, the stick and leaf insects, are a group of rather large herbivorous insects that is well known for their diversity of morphological adaptations facilitating imitation of parts of plants (Bedford 1978). One particular lineage of phamids is well known for its perfect mimicry of plant leaves: the walking leaves (Phylliidae). The phylogenetic relationships of phasmids in general (Bradler et al. 2015; Goldberg et al. 2015; Robertson et al. 2018; Büscher et al. 2018a; Glaw et al. 2019; Simon et al. 2019) and phylliids in particular (Bank et al. 2021) are subject to several studies based on morphological, as well as molecular data and corroborated the monophyly of Phylliidae on the one hand but recovered *Phyllium* as paraphyletic (Bank et al. 2021). *Chitoniscus* sensu lato in particular are rather short and stout leaf insects, which perfectly blend in with the foliage of the plants they dwell on (Fig. 1). Within their largely tree dominated, canopy dwelling habitats (Fig. 2) such small leaf insects are well hidden from potential predators. Due to their size and their, more or less, close distribution on New Caledonia and the Fiji islands, several species in past years were assigned to *Chitoniscus* sensu lato based on general morphological features. Large-scale phylogenetic studies on Phasmatodea repeatedly recovered *Chitoniscus* sensu lato as polyphyletic (e.g., Buckley et al. 2009; Bradler et al. 2015; Robertson et al. 2018; Forni et al. 2020; Bank et al. 2021). Apparently the two distributional centers (New Caledonia and Fiji) of these superficially similar types of leaf insects represent two separate lineages within Phylliidae, with the clade from Fiji always recovered as sister to the remaining phylliids, while the placement of the New Caledonia clade has been recovered within the remaining phylliids in different locations depending upon the tree topology recovered (Buckley et al. 2009; Bradler et al. 2015; Forni et al. 2020; Bank et al. 2021). Convergent traits are common within Phasmatodea (Buckley et al. 2009; Goldberg et al. 2015; Robertson et al. 2018; Büscher et al. 2018, 2019; Zeng et al. 2020) and particularly insular dwarfism is a common explanation for some small size convergences in vertebrates (e.g., Lomolino 2005) and also reported for insects (Hayashi 1990). Therefore, in light of the phylogenetic results, we aimed to review the assemblage of taxa assigned to *Chitoniscus* sensu lato to deliver morphological evidence for the two similar sized lineages, that have been considered congeneric since 1904 (Kirby 1904). The distribution, including the furthest latitude south reported for any phylliid, of these miniaturized phylliids indicates two distinct geographic clusters, that render the monophyly of *Chitoniscus* sensu lato even more unlikely. In turn, the two distributional centers of *Chitoniscus* sensu lato are not only separated, they are also interconnected by different phylliid taxa (Brock et al. 2021). Hence, the resemblance of the two lineages rather likely arises from secondary adaptation towards similar selective pressures, i.e., convergently. Specimens formerly assigned to *Chitoniscus* sensu lato were traced in museums, private collections, and other sources and were examined to evaluate the identities of the two groups of small-sized leaf insects. We challenged the present interpretation of *Chitoniscus* sensu lato with this dataset and compared these two lineages of small sized Phylliidae using morphological and distributional data. In the following we aimed to answer the research questions: i) Which morphological characters separate these two groups of leaf insects? ii) How can the observed molecular disjunct within *Chitoniscus* sensu lato be explained phenotypically and geographically? iii) How can this conflict be resolved taxonomically?

## Materials and methods

The following collection acronyms are used.

**AMNH**American Museum of Natural History, New York, USA;

**BPBM**Bishop Museum, Honolulu, Hawaii, USA;

**Coll DG** Private collection of Detlef Größer, Berlin, Germany;

**Coll RC** Private collection of Royce T. Cumming, California, USA;

**Coll SLT** Private collection of Stéphane Le Tirant, Québec, Canada;

**CUMZ**Cambridge University Museum of Zoology, Cambridge, United Kingdom;

**IAC** Institut Agronomique néo-Calédonien, La Foa, Nouvelle-Calédonie;

**IMQC** Insectarium de Montréal, Montréal, Québec, Canada;

**MNHN**Muséum National d’Histoire Naturelle, Paris, France;

**MZPW**Polish Academy of Sciences, Museum and Institute of Zoology, Warsaw, Poland;

**MHN** Muséum d’Histoire Naturelle, Geneva, Switzerland;

**NHMB**Naturhistorisches Museum, Basel, Switzerland;

**NHMUK**Natural History Museum United Kingdom, London, United Kingdom;

**NHMW**Naturhistorisches Museum Wien, Vienna, Austria;

**QM**Queensland Museum, South Brisbane, Australia;

**SDEI**Senckenberg Deutsches Entomologisches Institut, Müncheberg, Germany.

### Photography

Photographs of specimens deposited within the IMQC collection and Coll SLT were taken by René Limoges using a Nikon D850 DSLR camera (Nikon Corporation, Tokyo, Japan) with Nikon Micro-Nikkor 200mm f/4 lens on Manfrotto 454 micrometric positioning sliding plate (Manfrotto, Casolla, Italy). Lighting was provided by two Nikon SB-25 flash units with a Cameron Digital diffusion photo box (Henry’s, Vancouver, Canada).

Photographs from SDEI were taken by Arne Köhler (SDEI) using either a Nikon D7200 camera (Nikon Corporation, Tokyo, Japan) or Leica M205C-microscope (Leica Microsystems Inc., Buffalo Grove, USA). The stacking software used were Zerene Stacker (for the Nikon images; Zerene Systems LLC, Richland, USA) and Helicon Focus (for the Leica images; Helicon Soft Ltd., Kharkiv, Ukraine).

Photographs of specimens within the first authors collection (Coll RC) were taken by RC using a Canon 5D Mark II and a MP-E 65mm macro lens and stacked using Zerene Stacker (Zerene Systems LLC, Richland, USA). The eggs of *Trolicaphylliumsarrameaense* comb. nov. were photographed using a Nikon D3500 camera (Nikon Corporation, Tokyo, Japan) mounted on a Wild M3C stereomicroscope (Wild, Heerbrugg, Switzerland) and manually stacked. Adobe Photoshop Elements 13 (Adobe Inc., San Jose, USA) was used as post processing software.

Egg orientation terminology follows Clark (1978), and wing venation terminology follows Burt (1932) and Ragge (1955).

### Scanning electron microscopy (SEM)

Micrographs were obtained from dried samples, which were sputter coated with 10 nm gold–palladium. Overview images were obtained with a Hitachi TM3000 SEM (Hitachi High-technologies Corp., Tokyo, Japan) at 15 kV acceleration voltage using a rotatable specimen holder (Pohl, 2010). Detailed micrographs were obtained in the SEM Hitachi S4800 (Hitachi High-technologies Corp., Tokyo, Japan) at an acceleration voltage of 5 kV. Adobe Photoshop Elements 13 (Adobe Inc., San Jose, USA) was used as post processing software.

Morphological abbreviations (listed morphologically anterior to posterior)

**a1–a9>** antennomeres 1–9

**st** stridulatory file

**sr** stridulatory ridge

**ar** arolium

**cl** claw

**eu1–5** euplantula 1–5

**ta1–5** tarsomere 1–5

**ri** median ridgelike expansion

**C** costa

**Sc** subcosta

**R** radius

**R1** radius 1

**R2** radius 2

**Rs** radial sector

**R–M** radius to media crossvein

**M** media

**MA** media anterior

**MP** media posterior

**MP1** first media posterior

**MP2** second media posterior

**Cu+MA+MP** fused cubitus, media anterior, and media posterior

**Cu** cubitus

**CuA** cubitus anterior

**CuP** cubitus posterior

**Cu+1AA** cubitus and first anterior anal

**1A** first anal

**1AA–7AA** first–seventh anterior anal

**1PA–5PA** first–fifth posterior anal

## Results

The results of our morphological and biogeographic examination of *Chitoniscus* sensu lato strongly support the presence of at least two independent lineages, thereby necessitating the recognition of a new genus, *Trolicaphyllium* gen. nov., to accommodate the endemic New Caledonia taxa. The erection of this novel genus is supported by the identification of autapomorphic morphological features (discussed below) as well as years of molecular results recovering *Chitoniscus* sensu lato as polyphyletic (e.g., Buckley et al. 2009; Bradler et al. 2015; Robertson et al. 2018; Forni et al. 2020).

### Taxonomy

#### Trolicaphyllium gen. nov.

Taxon classificationAnimaliaPhasmatodeaPhasmatodea

AB27C687-1F25-53D1-8A10-E9810086754A

http://zoobank.org/FDDA1369-F249-451C-85A8-C1298F10EA58

##### Type species here designated.

*Phylliumbrachysoma* Sharp, 1898.

##### Taxonomic hierarchy.

Due to the general phylliid morphological features, and the consistent recovery of this clade nested within the greater phylliids in molecular studies, we herein place this genus within the tribe Phylliini Brunner von Wattenwyl, 1893.

##### Discussion.

The selected type species for this new genus is *Phylliumbrachysoma* Sharp, 1898 (= *Trolicaphylliumbrachysoma* (Sharp, 1898), comb. nov.) which was the first species described and is represented by two female syntype specimens collected on Lifou Island (Fig. 22). With the differentiation of the various species within this genus somewhat vague due to possible morphological variability, we felt the original species from a single known exact locality was the best choice as type species.

This new genus has been confused for decades with the similarly sized *Chitoniscus* Stål, 1875 sensu stricto from nearby Fiji due to their superficial similarities. All molecular phylogenies which have included both Fijian and New Caledonian samples have recovered these as polyphyletic (e.g., Buckley et al. 2009; Bradler et al. 2015; Robertson et al. 2018; Forni et al. 2020; Bank et al. 2021), with the *Chitoniscus* sensu stricto as sister to all other extant phylliids. Within the phylliid-wide phylogeny of Bank et al. (2021) the New Caledonian clade was recovered as sister to *Comptaphyllium*Cumming et al. 2019b with high support. Interestingly, few morphological features link these two genera, and it appears as though based upon morphological similarity, higher level relationships among the phylliids are difficult to ascertain. Only the intra-generic relationships appeared to agree readily when reviewing molecular and morphological data (Bank et al. 2021).

**Figure 3.**
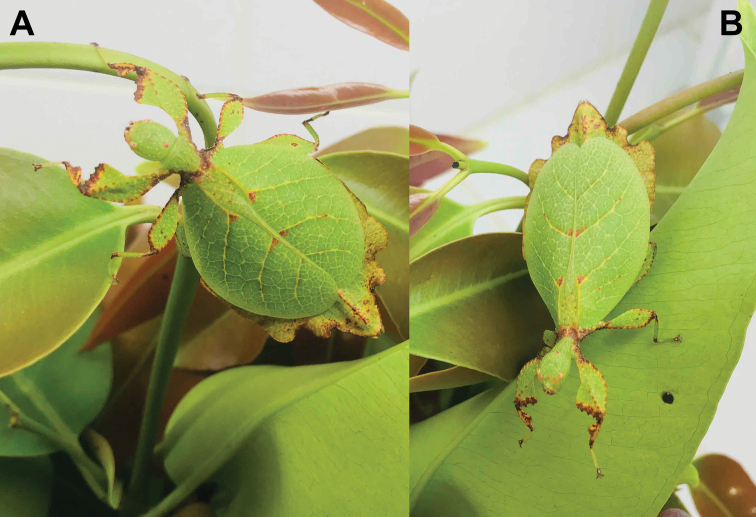
Adult female Trolicaphylliumcf.sarrameaense comb. nov. found feeding on *Syzygiumcumini* Sarramea county, near La Foa, January 2021 (recorded and photographed by Sylvie Cazeres (IAC)) **A** dorsoanterior, habitus **B** dorsal, habitus.

Little is presently known about the *Trolicaphyllium* gen. nov. ecology at the moment, as the only host plant records we have seen to date are from a *Ficus* sp. (recorded by Thierry Salesne; New Caledonia) and *Syzygiumcumini* (recorded by Sylvie Cazeres (IAC); Fig. 3). The only additional information we have regarding the ecology of this genus are short notes gleaned from specimen labels. In particular, “rainforest” appeared on many labels within the QM collection as noted by Geoff Monteith.

##### Morphological differentiation from *Chitoniscus* sensu stricto.

Features which liken these two genera together are their short length (ca. 40 to 60 mm) and broad bodies. Both genera have species which have smooth/tapered abdominal shapes or can be strongly lobed (within both males and females). The eggs of both species are small and lack pinnae therefore they superficially resemble each other.

However, when the finer details of these two genera are reviewed, the differences between them are significant (Table 1). A key to genera is not presented here as a thorough key was published within Bank et al. (2021) to all phylliid genera and can still be used to key to the *Chitoniscus* sensu lato couplet, at which point the features within Table 1 can then be used to differentiate these genera.

**Table 1.** Summary of morphological features for differentiating *Chitoniscus* Stål, 1875 sensu stricto from *Trolicaphyllium* gen. nov. as these two have been mistakenly associated for more than a century. Listed morphologically from the anterior to posterior.

Female	*Trolicaphyllium* gen. nov.	*Chitoniscus* Stål, 1875 sensu stricto
Antennae	Antennomeres III, VIII, and IX widened, broader than the antennomeres between; Fig. 4A.	Antennomeres III, VIII, and IX not widened, with similar diameter as the antennomeres between; Fig. 4B
Antennae: third antennomere	Broadened, sr not shifted anteriorly, sf meeting sr in anterior third; Fig. 5A. Sf long, ≥ 35 teeth; Fig. 5A	Not broadened, sr shifted anteriorly, sf meets sr at half of its length; Fig. 5B. Sf short, ≤ 30 teeth; Fig. 5B
Antennae: third antennomere (sf teeth)	Teeth with a smooth apex; Fig. 5C	Teeth with a weakly bilobed apex; Fig. 5D
Antennae: first antennomere	Dorsal surface without notable expansion, flush with anterior of the segment; Fig. 6A, C	Dorsal surface with expansion projecting anteriorly alongside the 2^nd^ antennomere, projecting beyond the anterior end of the segment; Fig. 6B, D
Protibial interior lobe	Always spanning the full length of the protibial shaft	Either absent or even in the most well-developed forms only on the proximal half, never fully spanning
Prescutum anterior rim sagittal spine	Spine and rim distinct, but not large; Fig. 7A. Rim and spine situated on the anterior margin, not strongly protruding posteriorly; Fig. 8A	Spine and rim prominent; Fig. 7B. Rim strongly protruding and angled posteriorly; Fig. 8B
Ventral coxae color	Green, similar shade as the remainder of the insect; Fig. 9A	Sky blue in color; Fig. 9B
Tegmina: R and M	R runs parallel with M until the split of Rs, at which point Rs bends away distinctly; Fig. 10A	R diverges steadily from M for the full length, therefore the split of the Rs is not a significant bend; Fig. 10B
Tegmina: R–M crossvein	R–M crossvein does not fade, but fully reaches to and connects with M; Fig. 10A	R–M crossvein thins and fades before reaching M; Fig. 10B
Terminal abdominal segment	Broad; almost two times as wide as long; Fig. 11A	Narrow; approximately as long as the greatest width; Fig. 11B
Cerci texture	Weakly granular/smooth; Fig. 11A	Heavily granular; rough textured; Fig. 11B
Tarsus	Euplantula 2 and 3 with ridgelike expansion along the entire tarsomere; Fig. 12B	Euplantula 2 and 3 without ridgelike expansion; Fig. 12B
**Male**		
Ocelli	Well developed; Fig. 13A	Absent; Fig. 13C, D
Protibial interior lobe	Always spanning the full length of the protibial shaft	Typically, absent or in well-developed forms only on the proximal half, rarely fully spanning and if so only as a thin lobe
Prescutum	Anterior margin more typical of a phylliid with the margin not strongly curved, making the prescutum appear less compacted; Fig. 13A, B	Anterior margin angled posteriorly, making the prescutum appear very stout; Fig. 13C, D
Alae: R split into R1 and Rs	Split is approximately ⅖ of the way through the wing; Fig. 14	Split is approximately halfway through the wing
Alae: MA and MP	Media anterior (MA) and media posterior (MP) veins fuse with the cubitus (Cu) at different locations along the cubitus and run fused to the wing margin; Fig. 14	Media anterior (MA) runs unfused to the wing margin; media posterior (MP) fades without fusing or reaching the wing margin
**Eggs**		
Operculum	Raised on the ventral end, not centrally raised, no pit, minimal granulation throughout; Fig. 15C	Centrally raised and with a pit in the center; Fig. 15F
General chorionic texture	Small spherical surface structures; Fig. 16A, B, also present on the micopylar cap Fig. 16C	Tuberculate chorionic surface, rough; Fig. 16D, E, pinnate micropylar cap; Fig. 16F
Microstructures	Mushroom-like smooth granula; Fig. 17B	Small pinnae arranged in ridges; Fig. 17E
**Freshly hatched nymph**		
Meso-, metafemoral coloration	Prominent white patch on the center of the exterior lobe and onto the femoral shaft; Fig. 18A	Mostly black in color, no prominent white patches; Fig. 18B
Mesonotum	Slender, posterior width similar to length; Fig. 18A	Stout, posterior width greater than length; Fig. 18B
Abdominal coloration	Abdomen black with the margins of segment II–IV and VI–IX green; Fig. 18A	Abdomen uniformly black, no green margins; Fig. 18B
**Distribution**	New Caledonia; Fig. 21	Fiji

##### Autapomorphic features.

Several morphological features unite the New Caledonian species and support monophyly of this clade within the phylliids. Within females, the euplantula 2 and 3 on the tarsus has the unique feature of a ridge-like expansion running along the entire tarsomere (Fig. 12B), a feature not seen in any other phylliids. Within males the alae venation (Fig. 14) is unique within the phylliids as the media anterior (MA) and media posterior (MP) veins fuse with the cubitus (Cu) at different locations along the cubitus and run fused to the wing margin (versus other phylliid genera which for example can have the MA and MP often fuse and run together to the wing margin, fuse with the Cu after first fusing together, never fuse and simply fade before reaching the margin, or fuse with the Cu at different locations but are also joined by the first radial (R1) and radial sector (Rs) and all run together to the wing margin). These autapomorphic features help to define the new genus *Trolicaphyllium* gen. nov. within the Phylliidae as well as differentiate them from the *Chitoniscussensu stricto.*

##### Generic characteristics.

The *Trolicaphyllium* gen. nov. are small to medium, with females ranging from 42.0 mm (in the smallest recorded *Trolicaphylliumerosus* comb. nov.; Redtenbacher 1906) to 60.0 mm long (in the largest *Trolicaphylliumsarrameaense*, comb. nov.; Größer 2008b), with males from 38.5 mm to 43.3 mm (in the smallest and largest *Trolicaphylliumbrachysoma*, comb. nov.; Größer 2008b). Typical general coloration is green, but in captivity orange/yellow has been induced (Fig. 19).

***Legs*.** Both sexes have interior tibial lobes on the protibiae which span the full length, lack lobes on the protibial exterior, and the meso-, metatibiae are simple, lacking both interior and exterior lobes. In both sexes the profemoral interior lobe is broader than the exterior lobe (distinctly so in males with a width almost two times that of the exterior lobe, sometimes in females the interior and exterior are almost even in width). In both sexes the profemoral interior lobe is generally only marked with three or four broadly spaced teeth (quite dulled in females; slightly more serrate in males). Both sexes have the interior meso-, and metafemoral lobes slightly broader or about even in width to the exterior lobes, but the interior lobes are always more prominently marked by serration.

**Figure 4.**
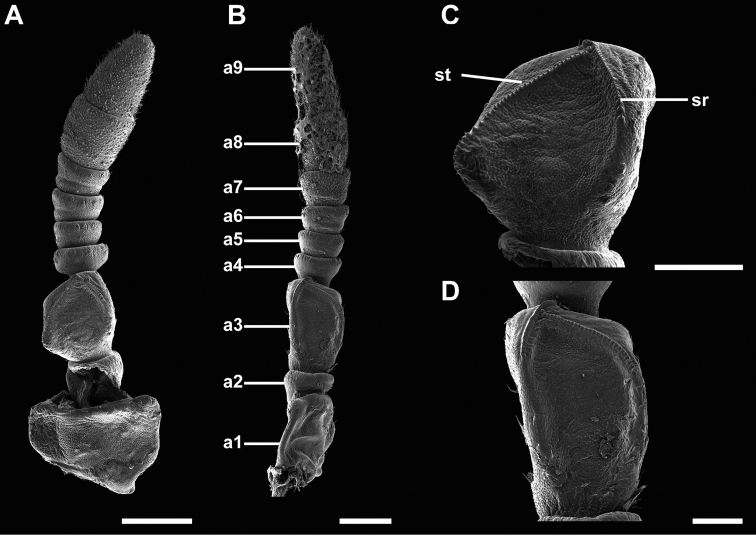
Scanning electron micrographs of female antennae **A, C***Trolicaphylliumsarrameaense* comb. nov. **B, D***Chitoniscus* sensu stricto **A, B** overview of the antenna, medial view **A** right antenna **B** left antenna **C, D** third antennomere. Abbreviations: a1–a9, antennomeres 1–9 st stridulatory file, sr stridulatory ridge. Scale bars: 300 µm (**A, B**), 200 µm (**C, D**).

**Figure 5.**
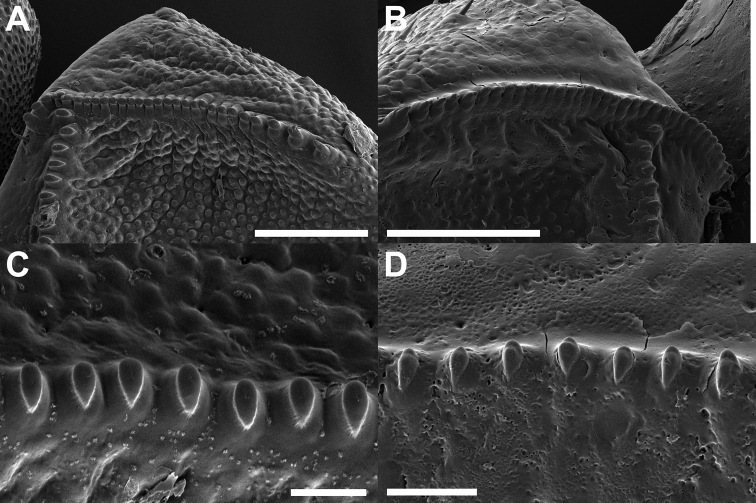
Scanning electron micrographs of female stridulatory organs **A, C***Trolicaphylliumsarrameaense* comb. nov. **B, D***Chitoniscus* sensu stricto **A, B** overview of stridulatory ridge **C, D** teeth of stridulatory file. Scale bars: 100 µm (**A, B**), 20 µm (**C, D**).

***Antennae*.** Females have antennae with nine segments with segments I, III, VIII, and IX notably broader than the other segments (Fig. 4A) and the stridulatory file has more than 35 teeth (Fig. 5A). Males have antennae which range from 23 to 26 segments with most segments covered in setae which are longer than the segment is wide.

**Figure 6.**
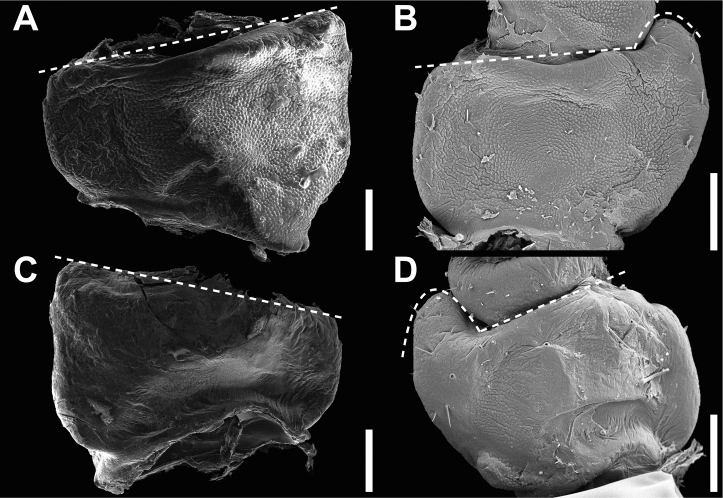
Scanning electron micrographs of female scapus (1^st^ antennomere) **A, C***Trolicaphylliumsarrameaense* comb. nov. **B, D***Chitoniscus* sensu stricto **A, B** overview of scapus, lateral view **C, D** medial view. Scale bars: 200 µm.

**Figure 7.**
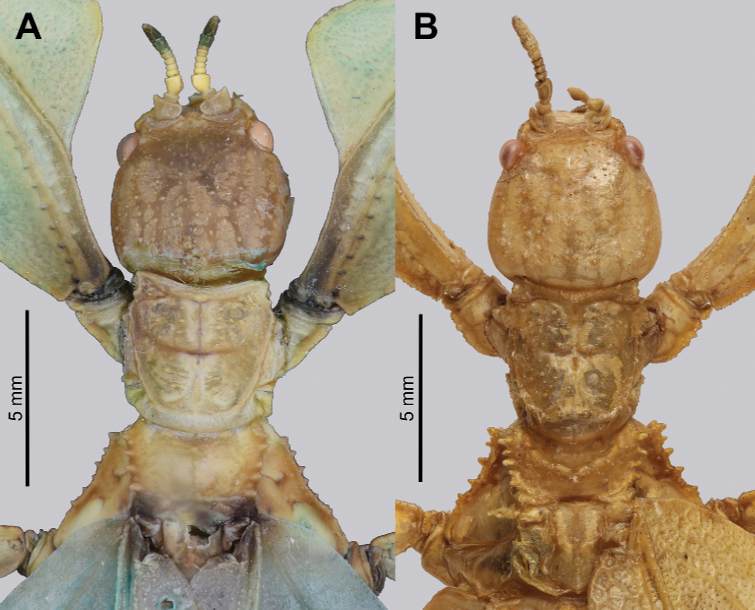
Details of the head through thorax, dorsal **A***Trolicaphylliumsarrameaense* comb. nov. (holotype) (DEI Hemimetabola, #100215) **B***Chitoniscus* sp. “Suva” (RC Coll 18-176).

***Head capsule*.** Males have well-developed ocelli (Fig. 13A), and both sexes have head capsules which are marked throughout by distinct granulation which is relatively evenly spaced and, in some cases, appears to be in slightly anterior to posterior rows (Figs 7A, 13A).

**Figure 8.**
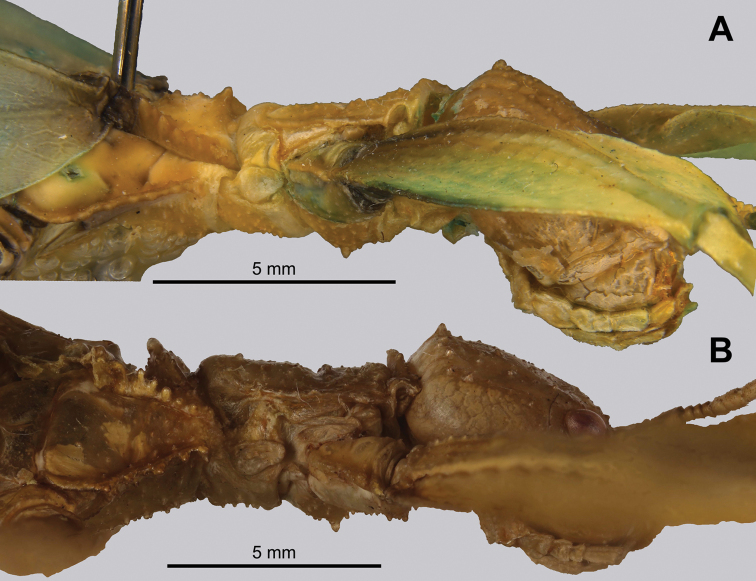
Lateral details of the head and thorax **A***Trolicaphylliumsarrameaense* comb. nov. (holotype) (DEI Hemimetabola, #100215) **B***Chitoniscus* sp. “Suva” (RC Coll 18-176).

***Thorax*.** The thorax is similar in both sexes with mesopleurae that are narrowly diverging from the anterior to the posterior and are marked with five to seven tubercles, occasionally with sparse setae interspersed (Figs 7A, 13A). In both sexes the prescutum is about two times wider on the anterior than long with lateral margins marked by six to eight tubercles, and a prescutum surface which is only slightly granular. When viewed laterally, both sexes have the prescutum anterior rim marked prominently with a raised sagittal spine and both have a prosternum which is prominently marked by a broad, warty tubercle (Figs 8A, 13B).

***Wings*.** Female tegmina are always long, reaching onto abdominal segments VII or VIII and male tegmina are moderate in length, reaching onto abdominal segment III. Females always have highly reduced alae, no more than just a nub (Fig. 22A). Male alae are always fully developed in an oval-fan configuration and reach onto abdominal segment IX (Fig. 14). Female tegmina have a subcoastal vein; radial vein which runs parallel with the media and splits into the first radial about halfway through its length and terminates in a radial sector and in a small radial to medial crossvein which does fully connect; a bifurcate medial vein; a bifurcate cubitus vein; and a first anal vein which fuses with the cubitus early on (Fig. 10A). Male tegmina have a subcoastal vein; radial vein which runs parallel with the media throughout the full length of the wing and branches into the first and second radial about one third and two thirds of the way through the wing length respectively and terminates as the radial sector; the media runs parallel with the radius and has two media posterior splits near the central area of the wing and terminates as the media anterior; the cubitus is unbranched; and there is a first anal which fuses with the cubitus early on (Fig. 14). Male alae (Fig. 14) have a costal vein running along the anterior margin; a subcostal vein which runs for about two thirds of the length and then fuses with the costal vein; the radial vein is bifurcate when it splits about two fifths of the way through the wing length where they diverge, run parallel, then converge sharply at the apex but don’t seem to reach the wing margin; the media is the most unique feature of the alae as it splits early on into the media anterior and posterior which run parallel until the media posterior fuses with the cubitus followed by the media anterior also fusing with the cubitus; the cubitus is fused with the first anterior anal for the majority of the length until the first anterior anal splits and runs to the wing margin; the cubitus, media anterior, and media posterior run fused to the wing margin; the anal veins are split into two groups, the anterior anals and the posterior anals (with seven anterior anals and five posterior anals).

**Figure 9.**
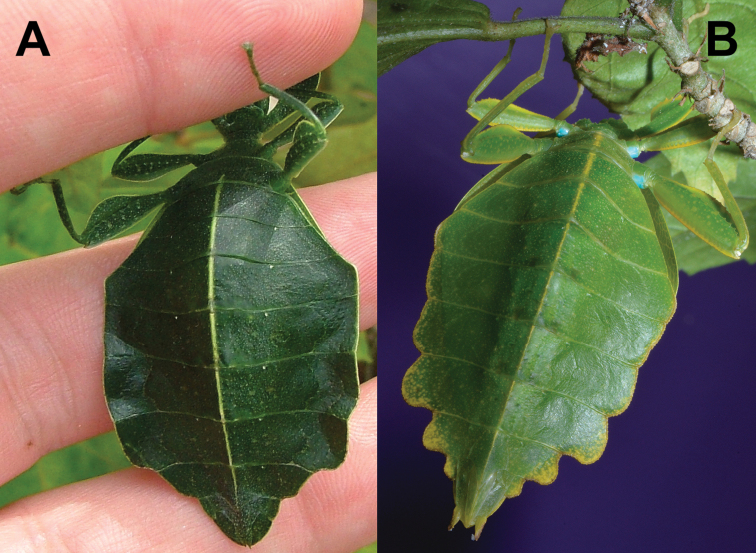
Ventro-posterior view of live females showing their exposed coxae coloration **A***Trolicaphylliumsarrameaense* comb. nov. taken by Thierry Salesne (New Caledonia) March 2011, in Vallée Pierrat, La Foa, Grand Terre **B***Chitoniscus* sp. “Suva” (RC Coll 18-176) live photograph taken by Thierry Heitzmann (Philippines).

**Figure 10.**
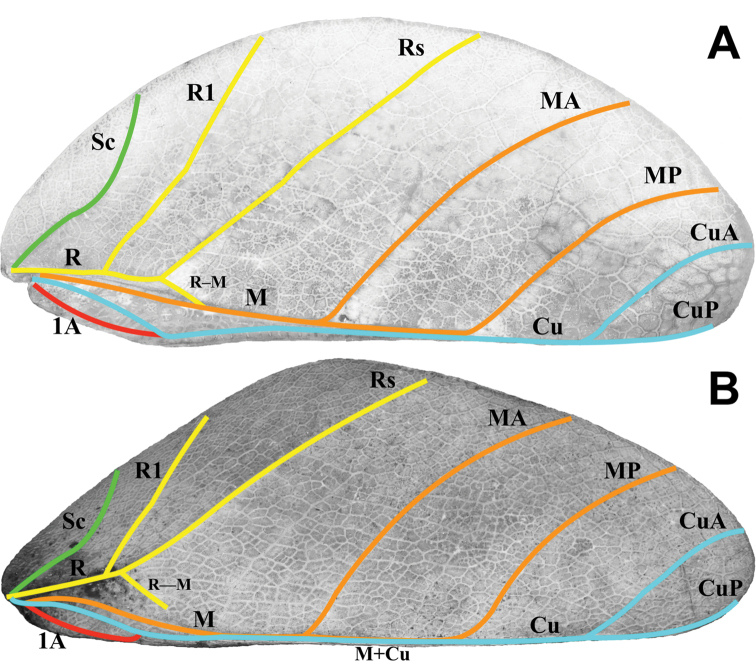
Female tegmina venation **A***Trolicaphylliumsarrameaense* comb. nov. (Coll SLT) **B***Chitoniscusfeejeeanus* from SDEI (#100213). Abbreviations: Sc (subcosta); R (radius); R1 (radius 1); Rs (radial sector); R–M (radius to media crossvein); M (media); MA (media anterior); MP (media posterior); Cu (cubitus); CuA (cubitus anterior); CuP (cubitus posterior); 1A (first anal).

**Figure 11.**
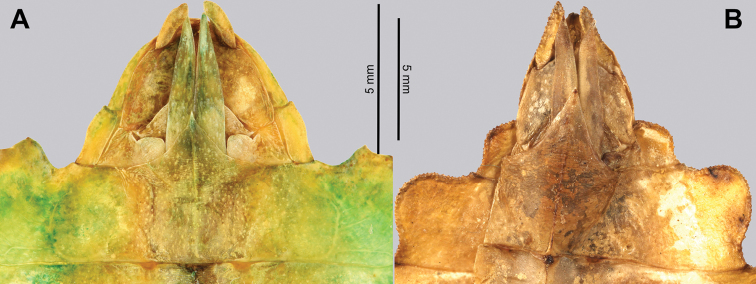
Female genitalia details, ventral view **A***Trolicaphylliumsarrameaense* comb. nov. (Coll SLT) **B***Chitoniscus* sp. “Suva” (RC Coll 18-176).

***Abdomen*.** Both sexes have variable abdominal shapes; females can range from spade-shaped to broad and boxy with prominently projecting abdominal lobes VII and VIII; males can be narrowly-ovoid and lack lobes to broadening until segment VII and converging with lobes. Female subgenital plate is short and stout with the apex reaching the anterior margin of the terminal abdominal segment and ending in a fine point; the gonapophyses VIII are long and slender, slightly exceeding the apex of the terminal abdominal segment; the cerci are relatively flat, marked sparsely with a granular surface with margins slightly marked with setae (Fig. 11A). Males have a broad, triangular vomer which is singularly pronged, hooking up into the paraproct.

***Egg.*** Egg morphology is only known at present from *Trolicaphylliumsarrameaense*, comb. nov. (Fig. 15A–C). Yasumatsu (1942) suggested an egg for *Trolicaphylliumbrachysoma* comb. nov. but the specimen the eggs were from was not collected on New Caledonia and likely represents a different genus and is unrelated to *Trolicaphyllium* gen. nov. based upon the illustration given. This general egg description is based upon examined material and on images of eggs from several sources all appearing to come from *Trolicaphylliumsarrameaense* comb. nov. females. Average length approximately 3 mm long. Eggs when viewed laterally are somewhat rectangular but with the dorsal surface slightly convex and longer than the ventral, giving the egg a slight bent appearance (Fig. 15B). Surfaces are marked throughout with shallow, irregular smooth patches which are accentuated by having darker coloration than the overall egg coloration. Eggs lack pinnae, but instead have small granulation scattered across the capsule which is most prominent and abundant along the capsule margins and notably sparse on the flat surfaces. The egg operculum is conically raised on the ventral margin only, not centrally raised like most phylliid eggs. The raised operculum is only about half as tall as wide and increases from the dorsal margin to the highest point on the ventral margin. The operculum apex has a similar granulation to that found on the capsule margins. Overall egg coloration variable, from a pale tan to light brown, or darker brown, with the pitting on the capsule darker in color and the granulation throughout lighter in color (Fig. 20).

**Figure 12.**
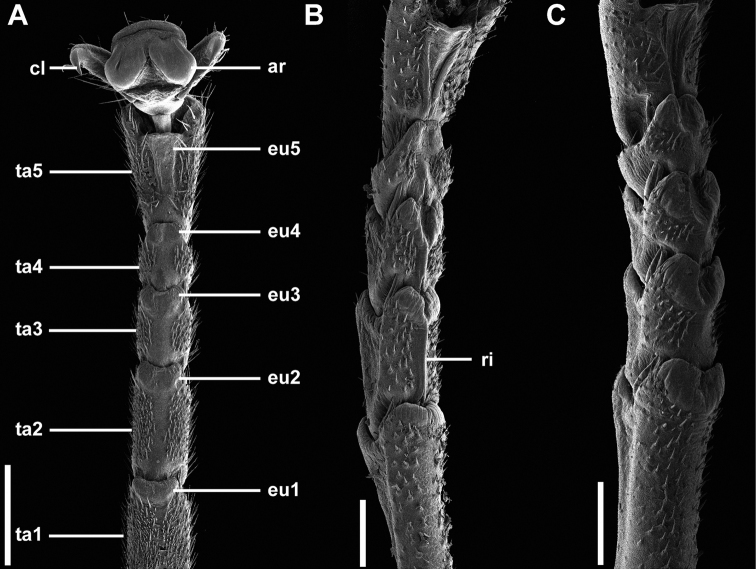
Scanning electron micrographs of tarsi **A, C***Chitoniscus* sensu stricto **B***Trolicaphylliumsarrameaense* comb. nov. **A** male, overview **B, C** females, tarsomeres 1–5. Abbreviations: ar, arolium, cl, claw, eu1–5, euplantula 1–5. Ta1–5, tarsomere 1–5, ri, median ridgelike expansion. Scale bars: 500 µm.

***Nymphs*.** Freshly hatched nymphs are known at present for *Trolicaphylliumsarrameaense* comb. nov. (Fig. 18A) but are unknown for the other *Trolicaphyllium* gen. nov. species. Therefore, a comparison between species is not possible at this time. This generalized description is based upon images of *Trolicaphylliumsarrameaense* comb. nov. shared by Detlef Größer (Germany). Body long and slender; profemora and protibiae with thin interior lobes but lack exterior lobes; meso- and metafemora with thin interior and exterior lobes; meso- and metatibiae simple, lacking lobes. The base coloration throughout the antennae, head, thorax, abdomen, meso- and metafemora is black. Profemora and all tibiae and tarsi are lighter colored, ranging from dark brown to tan/reddish. All joints between the tibiae and femora are marked with white. The meso- and metafemoral exterior lobes are marked with a medial white spot occupying approximately the central third of the lobe. The abdomen is slender and longer than the antennae, head, and thorax combined. Centrally the abdomen is black, but the margins of segments II–IV and VI–VIII are bordered with a lime green color.

###### New combinations

*Trolicaphylliumbrachysoma* (Sharp, 1898), comb. nov.

*Trolicaphylliumerosus* (Redtenbachher, 1906), comb. nov.

*Trolicaphylliumsarrameaense* (Größer er, 2008a), comb. nov.

**Etymology.***Trolicaphyllium* meaning “leaf that walks noiselessly”. This generic epithet is a compound of the Latinized name *Phyllium* the type genus for the family (from Greek *φυλλον*, -*ου* (*phyllon*, -*oy*) + -um; Poitout 2007), coupled with the prefix *tro lica* from the Drehu (Dehu) language phrase which means “walk noiselessly” (Tyron 1967). We wished to honor the original inhabitants of this area by using a local traditional language. We chose this name because these beautiful insects are so elusive and noiselessly living in the trees where they are often overlooked. This new genus is neuter in gender, following *Phyllium*.

**Figure 13.**
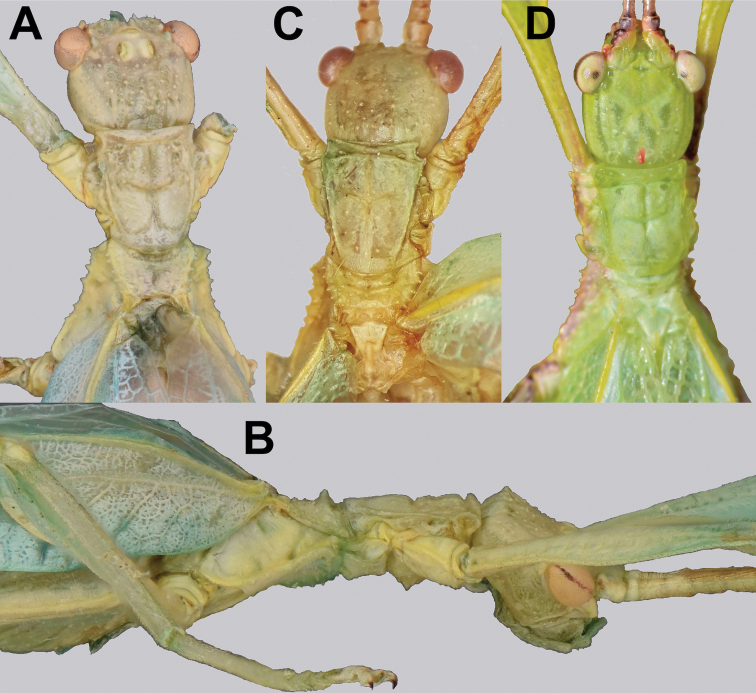
Details of male head through thorax **A, B***Trolicaphylliumsarrameaense* comb. nov., paratype male (#100214); photographs by Arne Köhler (SDEI) **C, D***Chitoniscus* sensu stricto **A, C, D** head through thorax, dorsal **B** head through thorax, lateral **C***Chitoniscus* sp. from the NHMUK, photograph by RC **D** live *Chitoniscus* sp. “Suva”, photograph by Thierry Heitzemann (Philippines).

**Distribution.** At present *Trolicaphyllium* gen. nov. specimens are only known from the country of New Caledonia, with records from Grande Terre, Lifou, Tiga, Maré, Ile de Bélep, and L’Île-des-Pins islands (Fig. 21). Likely other islands may also be suitable, but we have yet to locate specimen records from museums or observations.

#### Trolicaphyllium brachysoma

Taxon classificationAnimaliaPhasmatodeaPhasmatodea

(Sharp, 1898) comb. nov.

E9E27569-68C8-57E5-93DD-2094FFFD0480

**Figure 14.**
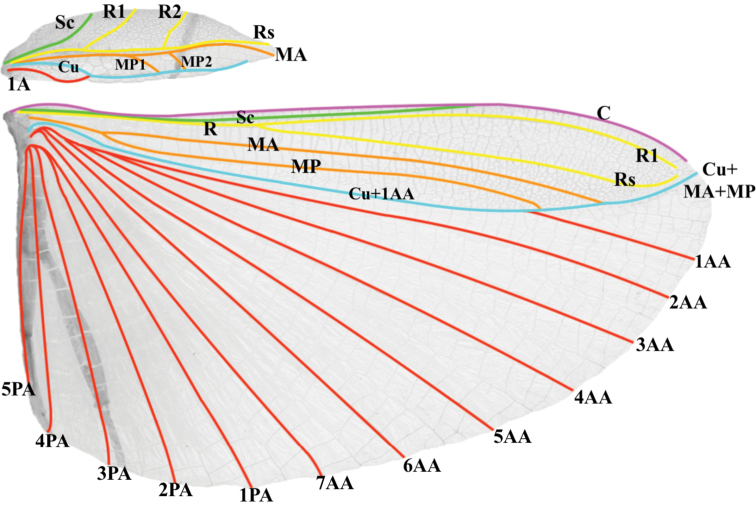
Male tegmina and alae venation Trolicaphylliumcf.brachysoma comb. nov. (Coll RC 16-094). Abbreviations: C (costa); Sc (subcosta); R (radius); R1 (radius 1); R2 (radius 2); Rs (radial sector); M (media); MA (media anterior); MP (media posterior); MP1 (first media posterior); MP2 (second media posterior); Cu+MA+MP (fused cubitus, media anterior, and media posterior); Cu (cubitus); Cu+1AA (cubitus and first anterior anal); 1A (first anal); 1AA–7AA (first–seventh anterior anal); 1PA–5PA (first–fifth posterior anal).

Figures 14 , 22 , 24 , 25

##### Material examined.

(35 ♀♀, 11 ♂♂, 2 unsexed nymphs): ***Syntypes*** (2 ♀♀): “Phyllium (Chitoniscus) brachysoma. Type D.S. Lifu. Dr. Willey. 1897” and “*Phylliumbrachysoma*. Type ex parte. D. Lifu. Willey. 1897” (CUMZ; Fig. 22). See Suppl. material 1 for additional specimens reviewed, their collection data, and depositories.

##### Remarks.

This was the first phylliid species recorded from New Caledonia and was therefore the first described *Trolicaphyllium* gen. nov. species, consequently, we here designate it as the type species for the new genus. Additionally, it was chosen as it has acceptably accurate collection data (Lifou Island; a rather small island instead of a general locality from the larger main island, which possibly contains several species) thereby removing some degree of possible confusion which could surround such old and difficult to distinguish specimens. This precise locality will allow future reviewers with adequate material sampled from numerous islands to identify species boundaries and determine if this species ranges across New Caledonia or if it is restricted to Lifou Island.

**Figure 15.**
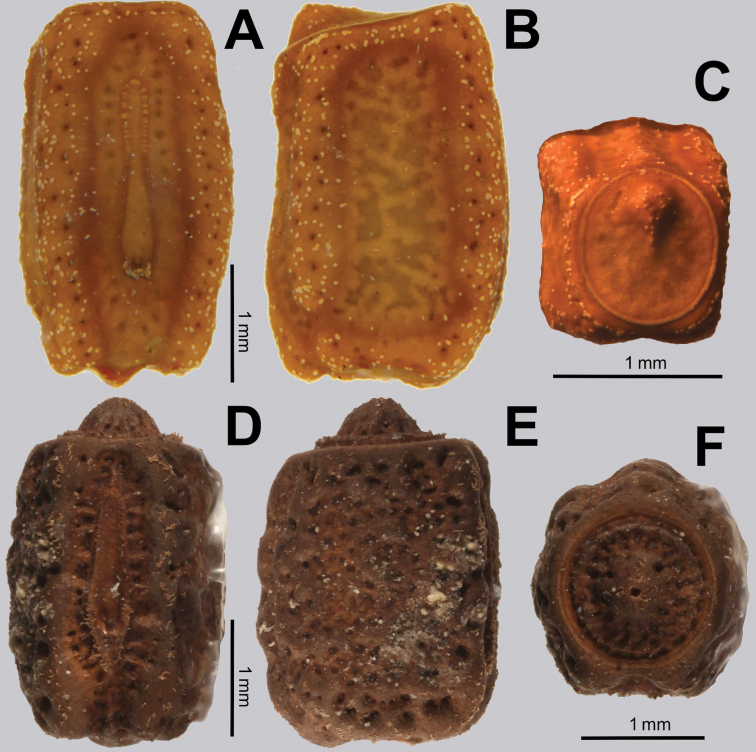
Comparison of *Chitoniscus* sensu stricto and *Trolicaphyllium* gen. nov. eggs **A–C***Trolicaphylliumsarrameaense* comb. nov., imaged by TB of eggs from Coll DG**A** dorsal **B** lateral **C** opercular (anterior) **D–F***Chitoniscus* sp. “Suva” (RC Coll 18-272) **D** dorsal **E** lateral **F** opercular (anterior).

**Figure 16.**
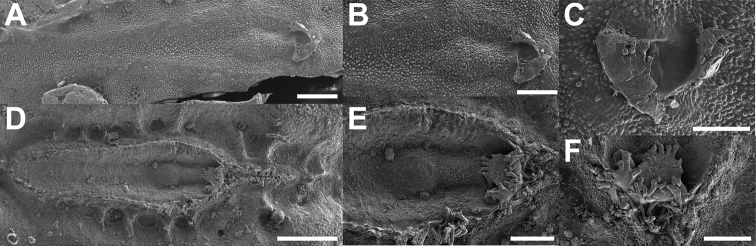
Scanning electron micrographs of specialized chorionic structures of the eggs **A–C***Trolicaphylliumsarrameaense* comb. nov. **D–F***Chitoniscus* sensu stricto **A, D** overview of micropylar plate **B, E** detail of micropylar plate **C, F** micropylar cap. Scale bars: 300 µm (**A, D**), 100 µm (**B, E, F**), 50 µm (**C**).

**Figure 17.**
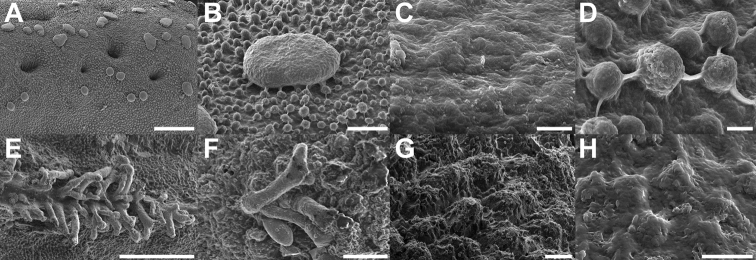
Scanning electron micrographs of chorionic microstructures on the eggs **A–D***Trolicaphylliumsarrameaense* comb. nov. **E–H***Chitoniscus* sensu stricto **A, B** mushroom-like granula **C, D, G, H** surface microsculpture **C** surface of the granula **D** exochorionic surface microstructures **E, F** pinnae. Scale bars: 100 µm (**A, E**), 20 µm (**B, F**), 10 µm (**G**), 5 µm (**D**), 3 µm (**H**), 1 µm (**C**).

**Figure 18.**
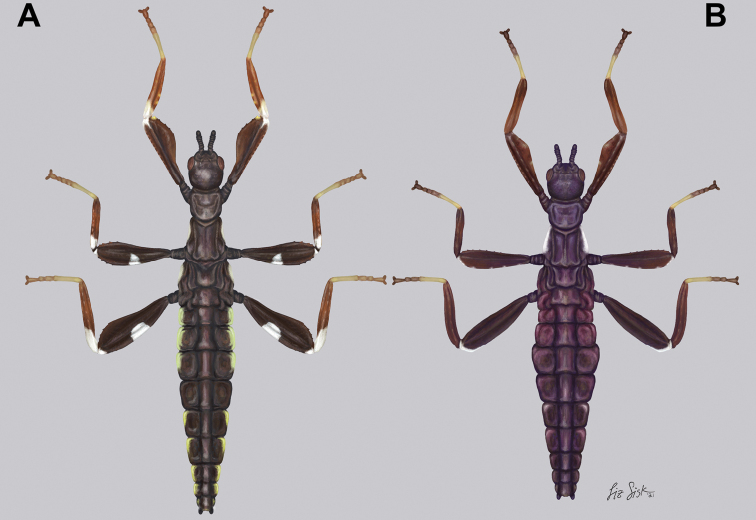
Illustrations of freshly hatched nymphs for comparison, dorsal habitus. Illustrations by Liz Sisk (USA). Nymph size is approximated to be relative to each other based upon the few photographs available but is only an estimate **A***Trolicaphylliumsarrameaense* comb. nov.; overall nymph length from head to tip of abdomen approximately 7 mm (Größer 2008b); illustration based upon photographs from Detlef Größer (Germany) **B***Chitoniscus* sp. “Suva’’ based upon images supplied by Mayk de Haan (Belgium).

**Figure 19.**
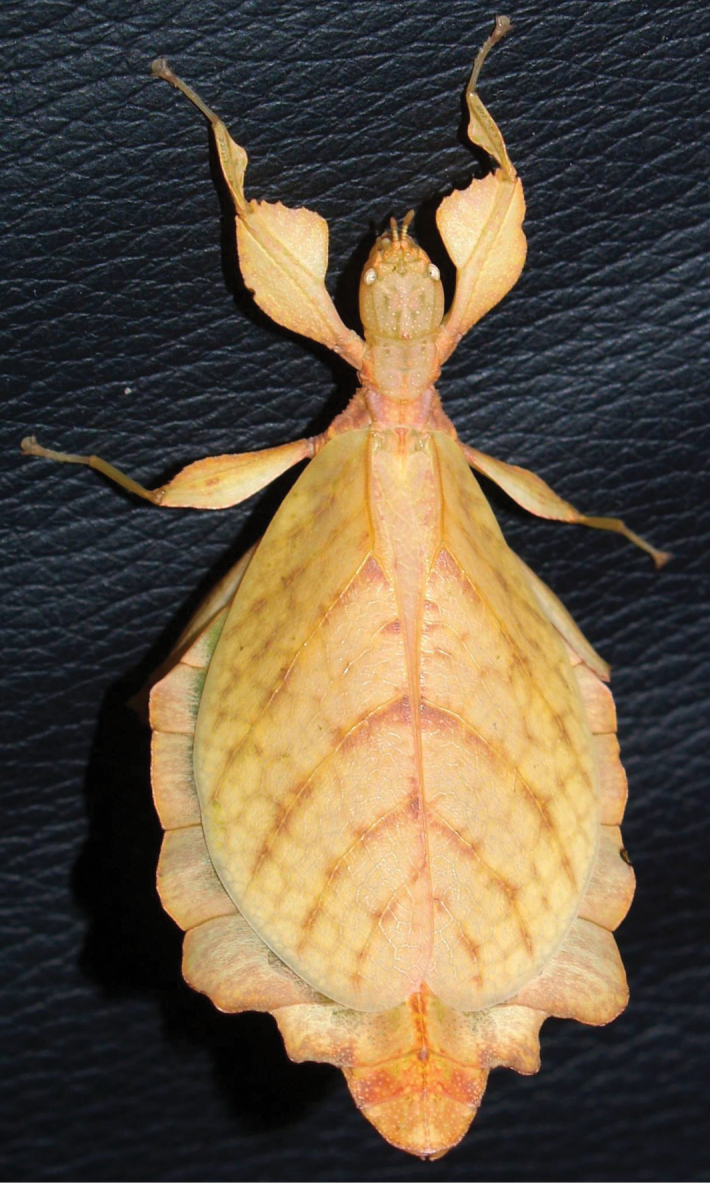
Captive bred *Trolicaphylliumsarrameaense* comb. nov. dorsal, habitus, female reared and photographed by Detlef Größer (Germany).

The syntype females were collected by Dr. Arthur Willey in 1897 while he was living on Lifou Island (Fig. 23; Sharp 1898). Dr. Willey was traveling and living in New Britain, New Hanover, eastern New Guinea, and Lifou Island between 1895 and 1897 in search of living Pearly Nautilus colonies which he could capture, collect eggs from, and rear through development in order to study their embryology (Wiley 1899; Kerr 1943). Although his years of expedition yielded many great discoveries of which he published prolifically, he was unfortunately unsuccessful in his primary goal of rearing eggs to maturity (Willey 1899). Willey lived on the west coast of Lifou Island on “Sandal Bay” (modern Santal Bay) from July 1896 to March 1897; and while no exact date was given with the syntype set of females, they are noted as being collected in 1897. Therefore, they are from the beginning of the year (January through March), and most likely from late January when a severe gale passed through the area (wreaking havoc on his Nautili traps; Kerr 1943) which likely knocked the phylliids from the canopy enabling them to be found by Dr. Willey. While males occasionally will fly to lights at night, females and nymphs are most often only found on the ground after storms when they are knocked from their typical canopy habitat and found lower (Brock and Hasenpusch 2003, 2015).

**Figure 20.**
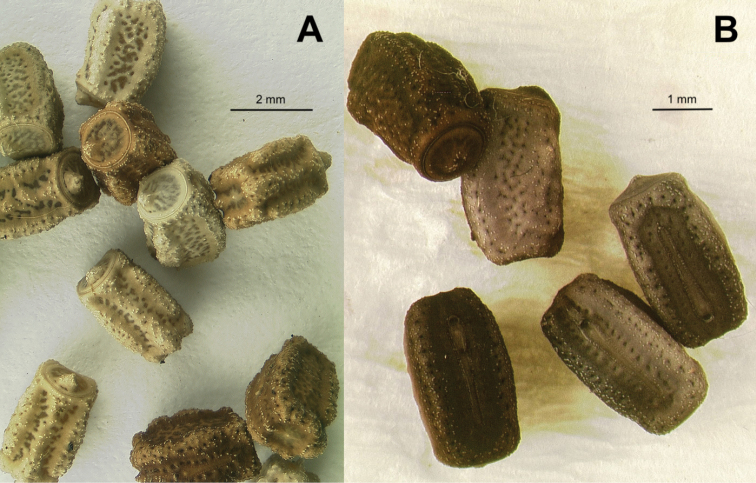
*Trolicaphylliumsarrameaense* comb. nov. eggs showing the variation in coloration, photographs by Sylvie Cazeres (IAC), eggs laid by females from Vallée Pierrat, Commune de La Foa **A** lighter colored eggs **B** first five eggs laid by the female from Figure 3.

No etymology was given by Sharp, but it can be assumed that he chose *brachysoma* to denote the size of the species, from the Greek words *brachy*- (short) and -*soma* (body).

##### Differentiation.

For female *Trolicaphylliumbrachysoma* comb. nov., one feature which appears to differentiate this species from the other two is the abdominal shape, which is lobeless, and tapered, giving them a spade-shaped appearance. It is worth noting however that in many phylliids abdominal shape is often a poor feature for differentiation as it is often variable within a single species (Cumming et al. 2020b), and even in the syntype set of two females, one female is notably more tapered (Fig. 22B) than the other (Fig. 22A). *Trolicaphylliumbrachysoma*, comb. nov. is about the same size as *Trolicaphylliumsarrameaense* comb. nov. (ca. 60 mm) which can differentiate them from *Trolicaphylliumerosus* comb. nov. which are notably smaller (ca. 40 mm).

Correctly matching up male and female phylliids is frequently a significant challenge due to their elusiveness in nature and sexual dimorphism and therefore opposite sexes can only be confirmed through molecular comparison or captive rearing (Cumming et al. 2020c; Cumming et al. 2021). Unfortunately, we have yet to confidently confirm a male *Trolicaphylliumbrachysoma* comb. nov. and therefore, at this time can only illustrate presumed males (Fig. 24) which follow the morphology of the female by having a smooth tapered abdomen and falling within an appropriate size range for a potential male (38 to 43 mm). *Trolicaphylliumerosus* comb. nov. has no presumed male records we are aware of (as no possibilities have been located in collections which are small enough to represent a male of this species) but based upon female size the male *Trolicaphylliumerosus* comb. nov. is likely rather small.

##### Distribution.

**Figure 21.**
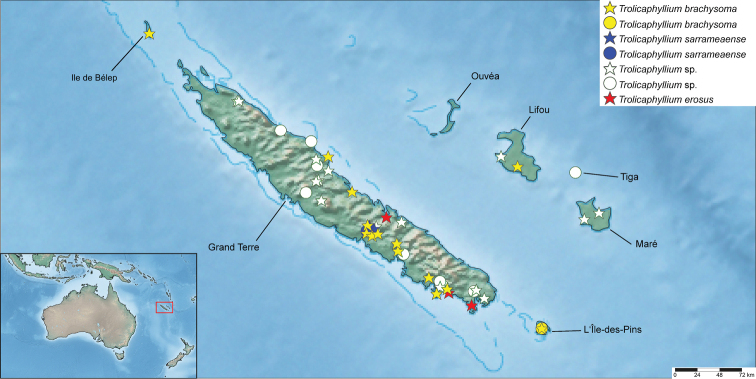
Distribution map noting all presently known *Trolicaphyllium* gen. nov. records which could be traced and accurately noted. See Suppl. material 1 for full details for all records presented. Stars indicate a record based upon a specimen, circles represent a record based upon a photographic observation. Produced with SimpleMappr (Shorthouse, 2010).

The type locality for this species is Lifou island, but *brachysoma*-like specimens with the tapered, lobeless abdomen have been found on Grande Terre (Fig. 21) and L’Île-des-Pins (Fig. 25) as well. Additionally, within the MNHN there is a female which was collected on Ile de Bélep, which is the only phylliid record we have seen from this island, and we only tentatively note this specimen as this species as it has slight lobes on the abdomen and could not be examined in person. Hopefully future molecular analyses with material from multiple islands will reveal if these are all one species or several.

**Figure 22.**
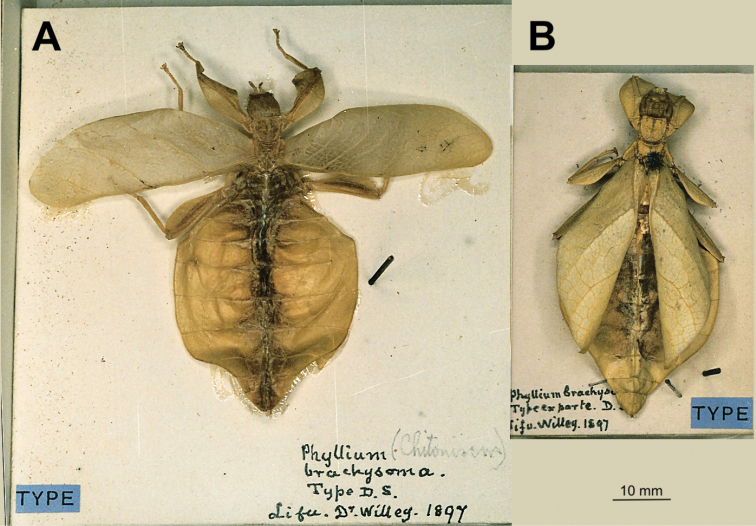
Syntype females of *Trolicaphylliumbrachysoma* comb. nov. the herein designated type species for the new genus. Photographs by Paul Brock (United Kingdom) of the set within the CUMZ**A** dorsal habitus with tegmina spread; note the lack of developed alae **B** dorsal habitus with tegmina closed.

**Figure 23.**
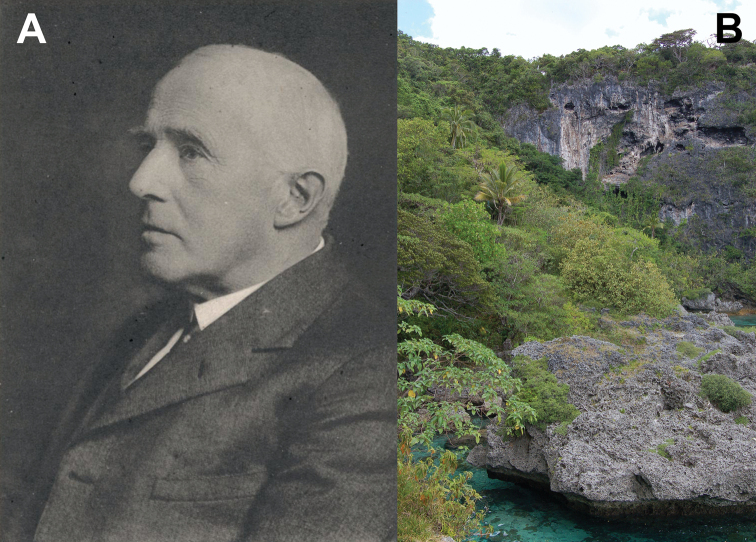
**A** Arthur Willey (1867–1942), collector of the *Trolicaphylliumbrachysoma* comb. nov. females in 1897 from Lifou Island while searching for the Pearly Nautilus; image used from the public domain due to expired copyright; from Kerr (1943)**B** Lifou, cliffs on the north end of the island slightly farther north of where Willey was stationed 1896–1897; photograph by user Bahnfrend in November 2007; used under Creative Commons 3.0 (CC BY-SA 3.0) (https://commons.wikimedia.org/wiki/File:Jokin,_Lifou,_2007_(4).JPG).

**Figure 24.**
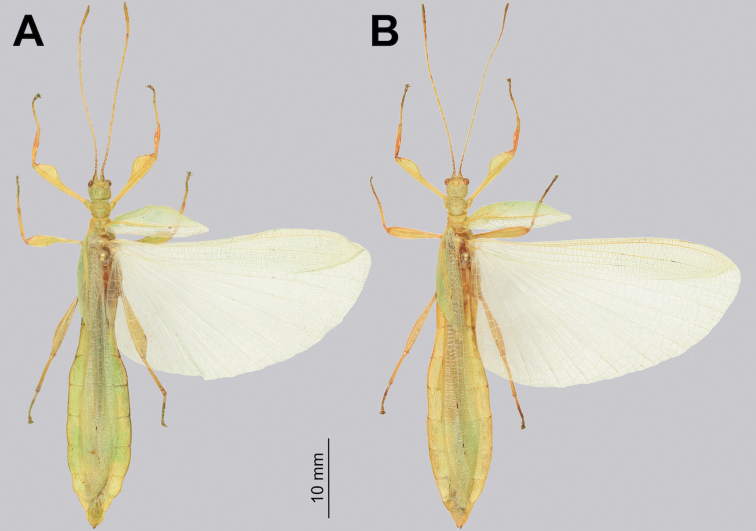
Dorsal habitus of male Trolicaphylliumcf.brachysoma comb. nov. **A** Bouloupari Commune, March 2013 (Coll RC 16-095) **B** Sarramea Commune, February 2009 (Coll RC 16-094).

**Figure 25.**
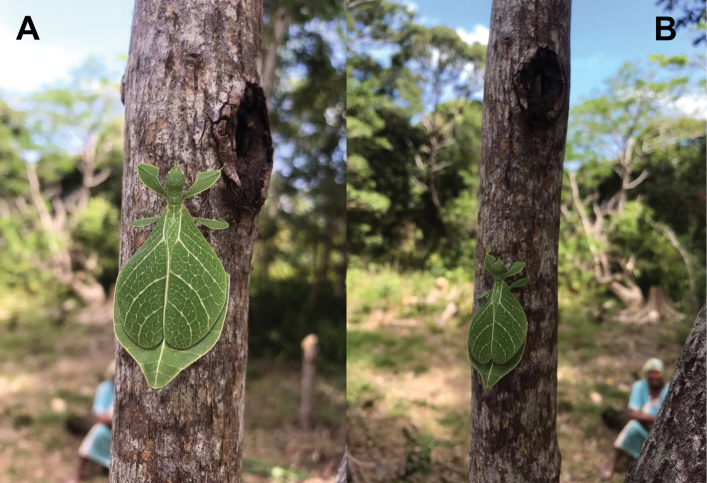
Trolicaphylliumcf.brachysoma comb. nov. adult female observed on L’Île-des-Pins by Patrice Kaateu (New Caledonia) in November 2020 **A** dorsal, habitus **B** same individual as in **A** but zoomed out to give scale/perspective.

#### Trolicaphyllium erosus

Taxon classificationAnimaliaPhasmatodeaPhasmatodea

(Redtenbacher, 1906) comb. nov.

D7B90DCE-8C7D-5198-9FE6-7A1E3040578B

Figures 26 , 27

##### Material examined.

**Figure 26.**
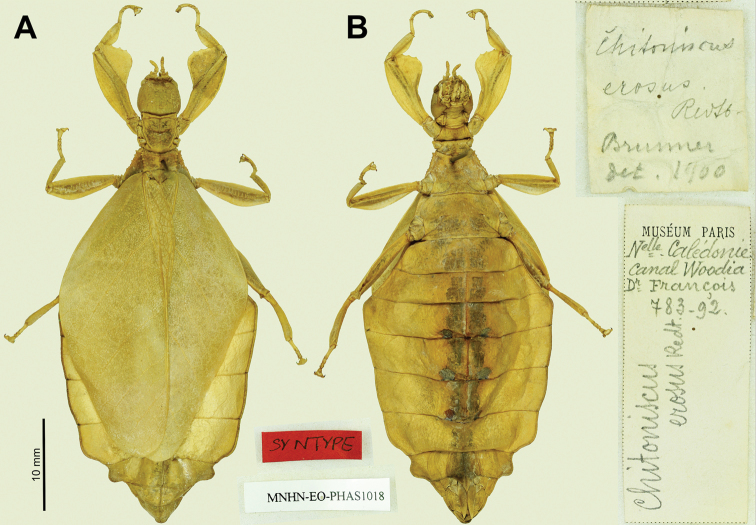
*Trolicaphylliumerosus*, comb. nov. syntype specimen from the MNHN collection. Photographs by: Emmanuel Delfosse (MNHN) **A** habitus, dorsal **B** habitus, ventral. Associated data labels inserted between and to the right.

**Figure 27.**
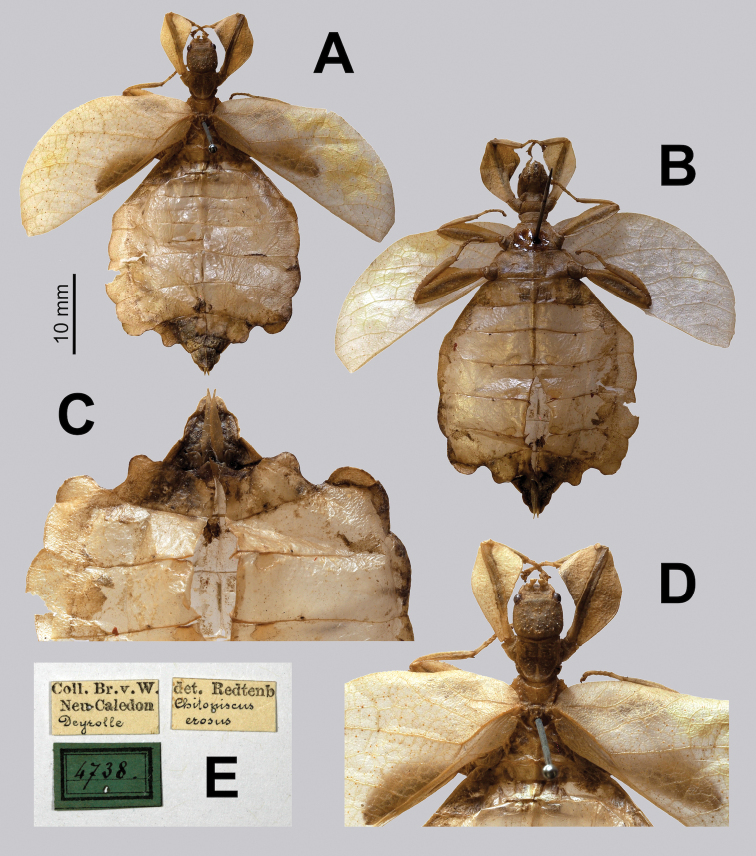
*Trolicaphylliumerosus* comb. nov. syntype female originally from the collection of Brunner von Wattenwyl, then passed on to Redtenbacher, now deposited in NHMW. Photographs by Harald Bruckner (NHMW) **A** habitus, dorsal (scale bar only representative of this image) **B** habitus, ventral **C** genitalia detail, ventral **D** details of the front legs, head, and thorax, dorsal **E** specimen data labels.

(10 ♀♀): Syntypes (2 ♀♀): “Syntype; MNHN-EO-PHAS1018; Museum Paris Nelle Caledonie Canal Woodia Dr. Francois 783-92. *Chitoniscuserosus* Redt.; *Chitoniscuserosus* Redtb. Brunner det. 1900” (MNHN; previously stored in alcohol, Fig. 26); “Coll. Br.v.W. Neu Caledon Deyrolle; det. Redtenb *Chitoniscuserosus*; 4738” (NHMW; Fig. 27).

See Suppl. material 1 for additional specimens reviewed, their collection data, and depositories.

##### Remarks.

This was the second species described from New Caledonia and was described by Redtenbacher (1906) where he gave little to differentiate the species from *Trolicaphylliumbrachysoma* comb. nov. except for the abdominal shape and the overall size as being smaller.

Within the original description by Redtenbacher the number of syntypes was vague, but at least three are explicitly stated as “New Caledonia (Coll. M., Mus. Paris); New Guinea (Mus. Budapest)” (Redtenbacher 1906). At least one syntype was within Redtenbacher’s own collection (noted as “Coll. M.” within his work) and eventually his collection was deposited in Vienna where it resides today, and the specimen could be traced (Fig. 27; Kaltenbach 2003). Additionally, he states at least one syntype from the “Mus. Paris” which has also survived and was traced (Fig. 26).

An additional syntype was explicitly noted as a nymph within the Budapest Museum (with the wording suggesting that there is only one syntype within that collection). Unfortunately, a fire during the Hungarian Revolution of 1956 destroyed the Budapest syntype along with many of the museum’s important type specimens (Brock 1998; Földvári and Papp 2007; Sabaj 2020). This syntype within the Budapest Museum was noted as being a nymph female from New Guinea with large broad forelegs reminiscent of “*Phylliumpulchrifolium* Serv.” (Redtenbacher 1906). Based on this information it is very likely that this was not actually a *Trolicaphyllium* gen. nov. specimen as this genus is restricted to New Caledonia and is only known to have rounded profemoral lobes. Instead, this nymph specimen was more likely a female *Nanophyllium* Redtenbacher, 1906 of which certain species can be small and reminiscent of *Trolicaphyllium* gen. nov. or *Chitoniscus* specimens but have broader profemoral exterior lobes (for example *Nanophylliumchitoniscoides* (Größer, 1992)).

**Figure 28.**
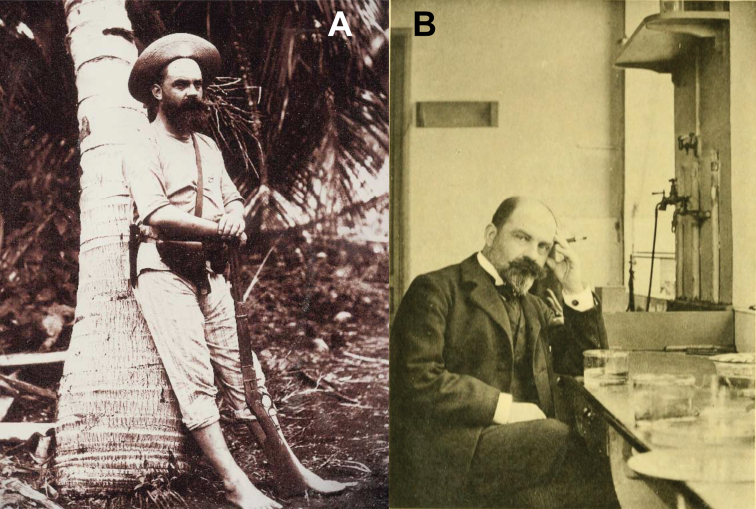
Philippe François the collector of one of the syntype *Trolicaphylliumerosus* comb. nov. females from within the MNHN collection **A** photographed in Vanuatu sometime between 1888 and 1895; used with permission from Tajan (Tajan 2002) **B** later in life back home in Paris, France where he reviewed many of the specimens he brought back from his expeditions (Bulletin Scientifique 1909).

Within the MNHN there is an adult female syntype specimen collected by Philippe François (Fig. 28) from Canal Woodia (canal de Woodin), a common shipping path which runs between Ile Ouen and the south coast of Grande Terre (Bernard 1894). The MNHN type database has the specimen reported as being collected sometime in 1892 (based upon the specimen label “-92”), however, this record number appears to not correspond to the collection year as Francois was not in Oceania 1892. By all accounts, François was actively researching in Oceania from 1888–1891, was back in France in 1892, and then returned to Oceania from 1893–1895 (Bulletin Scientifique 1909; Dewarumez 2011). We believe that this date on the label instead corresponds to the date the specimens were received by the museum and was instead collected by Francois between 1888 and 1889 when he passed through Canal Woodia (canal de Woodin) several times during his first journey to New Caledonia to study the biology of coral reefs (Bouyssi 1997).

Interestingly, Redtenbacher only appears to have measured one specimen of the type series, the one within his own collection (now within the NHMW), most likely he had seen the Paris and Budapest specimens prior and did not measure them while reviewing them. Harald Bruckner (NHMW) measured the single syntype within their collection and it agrees with the measurements recorded by Redtenbacher (Fig. 27). Interestingly, the smaller overall body length (42.0 mm) appears to be somewhat artificial as the abdomen is significantly excavated and somewhat ballooned outward, pulling the abdomen shorter (Fig. 27C). This is supported by the observation that the other features of this specimen (such as the tegmina length; 27 mm) are actually closer to average sizes for other *Trolicaphyllium* gen. nov. specimens of other species. Realistically it seems this specimen if it were naturally flat might actually be closer to a range of 50 mm, and not significantly smaller after all.

No etymology was given in the original description, but it can be assumed that Redtenbacher was referencing the lobed abdominal shape with *erosus*, from the Latin *e*- (out/away from) and -*rosus* (gnaw/peck), noting that the leaf-like body appears gnawed on, giving it the undulating appearance.

**Figure 29.**
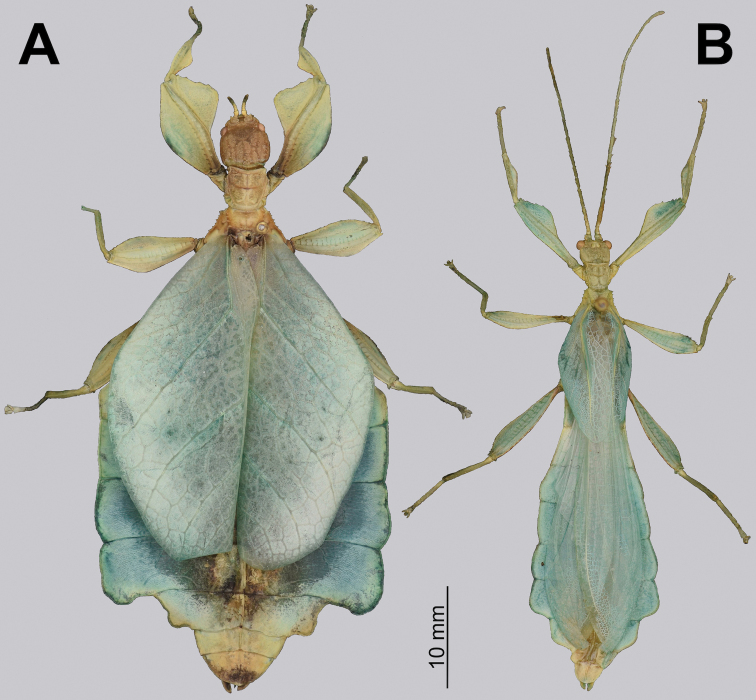
Dorsal habitus *Trolicaphylliumsarrameaense* comb. nov. **A** holotype female (#100215) **B** paratype male (#100214). Photographs by Arne Köhler (SDEI). Scale bar: 10 mm.

##### Differentiation.

To date this appears to be the least known species on New Caledonia as we have not been successful in tracing any additional females which fall within this small size range of ca. 40 mm beyond one of the syntypes. All specimens examined so far appear to belong to the other species with their larger size (ca. 50–60 mm). In fact, it is only this smaller size which we have found as useful for differentiation as the other features match up with the prominently lobed *Trolicaphylliumsarrameaense* comb. nov. females. The additional specimens identified as *Trolicaphylliumerosus* comb. nov. within the MNHN are larger than the syntype within the NHMW and several are rather morphologically similar to *Trolicaphylliumsarrameaense* comb. nov. due to their larger size and lobed abdomen but are at this time tentatively left identified as *Trolicaphylliumerosus* comb. nov. within the collection.

Additionally, we have yet to locate any possible male *Trolicaphylliumerosus* comb. nov. as all males located were much too large and appear to belong to the other two species.

##### Distribution.

The only specimen we have located with detailed locality information is the syntype female from the MNHN collection which has the additional information of “Canal Woodia” which is a small canal between Ile Ouen and the south coast of Grande Terre (Fig. 21). The additional non-type specimens from the MNHN are included within the distribution map, but only tentatively as they are slightly larger than the measured syntype from NHMW and they have variable abdominal shapes, some more strongly lobed than others.

#### Trolicaphyllium sarrameaense

Taxon classificationAnimaliaPhasmatodeaPhasmatodea

(Größer, 2008) comb. nov.

C3C5916A-9C50-5F26-9B98-9AA2B8BFBEF9

Figures 1 , 3 , 4A , 4C , 5A, C , 6A, C , 7A , 8A , 9A , 10A , 11A , 12B , 13A, B , 15A–C , 16A–C , 17A–D , 18A , 19 , 20 , 29

##### Material examined.

(8 ♀♀, 9 ♂♂, 3 eggs): Holotype and paratypes examined: 1 ♀, 1 ♂, 3 eggs: “Chitoniscus, sarrameaensis, Neu Kaledonien, Sarramea, Sep. 2006, det.Größer” (SDEI: HT♀, DEI Hemimetabola #100215; PT♂, DEI Hemimetabola #100214; PT eggs, DEI Hemimetabola #100216); (SDEI; Figs 7A, 8A, 13A, B, 29).

See Suppl. material 1 for additional specimens reviewed, their collection data, and depositories.

##### Remarks.

As it was only described in 2008, this was the most recently described species from New Caledonia with type material originally collected by Sigetake Suzuki in 2004 from Sarramea (Größer 2008a). Within the original description this species was not explicitly compared with the sympatric and morphologically very similar *Trolicaphylliumerosus* comb. nov. but was instead only differentiated from *Chitoniscuslobipes* Redtenbacher, 1906, where most features given for differentiation were simply the features we discuss above as significant for differentiating the two genera.

Other lobed specimens have been recovered from throughout New Caledonia, but unfortunately most have been nymphs (such as several from within the QM collection) and therefore they could not be confidently identified as *Trolicaphylliumsarrameaense* comb. nov. or as *Trolicaphylliumerosus* comb. nov. nymphs. Unfortunately, in Größer (2008b) the key to species of *Chitoniscus* sensu lato tried to use the female tegmina radial and media venation pattern to differentiate species, but mixed up the species. Within the key *Trolicaphylliumerosus* comb. nov. and *Trolicaphylliumbrachysoma* comb. nov. (from New Caledonia) instead key out as the Fijian population and *Chitoniscuslobiventris* (Blanchard, 1853) and *Chitoniscuslobipes* Redtenbacher, 1906 (from Fiji) key out as the New Caledonian population. We have reviewed the type specimen photos available on the Phasmid Species Files (Brock et al. 2021; http://phasmida.speciesfile.org) as well as numerous museum specimens, and always the female tegmina venation allowed accurate distinction of these two genera. Even if you look past this inaccuracy within the key, unfortunately no additional features can be gleaned from the further couplets to allow differentiation of *Trolicaphylliumsarrameaense* comb. nov. from *Trolicaphylliumerosus* comb. nov. (as the further couplets discuss abdominal shape, which in these two species is identical/variable). At this moment in time, we still lack significant details about the population of *Trolicaphyllium* gen. nov. on Grande Terre as material is limited and molecular data has yet to be compared across a wide sampling on the island. With phylliid abdominal shapes sometimes rather variable within a single species, this makes us wonder if *Trolicaphylliumsarrameaense* comb. nov. is in fact a valid species, or simply a synonym of *Trolicaphylliumerosus* comb. nov. which was described more than 100 years previously from the same island. Our examination of all type specimens which could be traced has not yet revealed additional features for morphological differentiation besides the overall size of these two species. Hopefully, future molecular analyses from across New Caledonia will reveal if there are several species present on Grande Terre or if it is simply a single species which can range in size from smaller (ca. 40 mm; *Trolicaphylliumerosus* comb. nov.) to larger (ca. 60 mm; *Trolicaphylliumsarrameaense* comb. nov.). It is due to this lack of sound molecularly based evidence and the propensity for phylliids to be morphologically variable that we refrain from synonymizing *Trolicaphylliumsarrameaense* comb. nov. with *Trolicaphylliumerosus* comb. nov. as we feel a significant decision such as this should be based upon a solid foundation. If future molecular analyses reveal that there is only a single morphologically variable species of *Trolicaphyllium* gen. nov. on Grande Terre based upon a sampling throughout the island, then we feel a synonymization will be necessary, but not until that time.

The etymology given in the original description is that this name is a toponym, named after the type locality, Sarramea, New Caledonia (Größer 2008a). The original combination was with the masculine genus (*Chitoniscus*) and therefore in order for the species name to agree in gender with our newly erected genus, the spelling of “*sarrameaensis*” is changed to the neuter gender “*sarrameaense*”.

##### Differentiation.

Females can be differentiated from *Trolicaphylliumbrachysoma* comb. nov. based upon abdominal shape, as *Trolicaphylliumbrachysoma* comb. nov. are considered to have a spade-shaped abdomen, with smooth margins, versus *Trolicaphylliumsarrameaense* comb. nov. which has a broad abdominal shape with parallel sides, ending in lobed segments VII and VIII. From *Trolicaphylliumerosus* comb. nov. the only feature we have been able to identify as useful is the overall length, with *Trolicaphylliumerosus* comb. nov. ca. 40 mm long versus *Trolicaphylliumsarrameaense* comb. nov. ca. 60 mm long.

Unfortunately, males of *Trolicaphylliumbrachysoma* comb. nov. and *Trolicaphylliumerosus* comb. nov. have never been confidently confirmed through breeding or molecular comparison. Based upon the confidently confirmed male/female *Trolicaphylliumsarrameaense* comb. nov. however, we expect that the male morphology should mirror the female morphology. Most likely the male *Trolicaphylliumbrachysoma* comb. nov. will lack prominent abdominal lobes and the male *Trolicaphylliumerosus* comb. nov. will have distinct lobes to match with their female counterparts. Based upon the female *Trolicaphylliumerosus* comb. nov. smaller size, we expect that the male must also be rather small, which could likely be used as a feature for differentiation.

##### Distribution.

To date we have only confirmed adult specimens and observations which are the correct morphology and size of *Trolicaphylliumsarrameaense* comb. nov. from central Grande Terre (Fig. 21). We have however seen nymph specimens which had characteristically lobed abdomens which may represent this species from other locations on Grande Terre, so we expect this species may be widespread throughout the island.

Within the MZPW collection there is a pair of *Trolicaphylliumsarrameaense* comb. nov. specimens with the data of simply “Lifu”, which if true could lend credibility to the hypothesis that perhaps these species are all variable in their abdominal shape (if there is only one species present on Lifou island), but as these are antique and give no other data, we do not take these as highly credible, and therefore exclude this record from further discussion and they are not included within the distribution map (Fig. 21). Or it is possible Lifou island holds several morphologically different species.

## Discussion

### Generic identity

**Figure 30.**
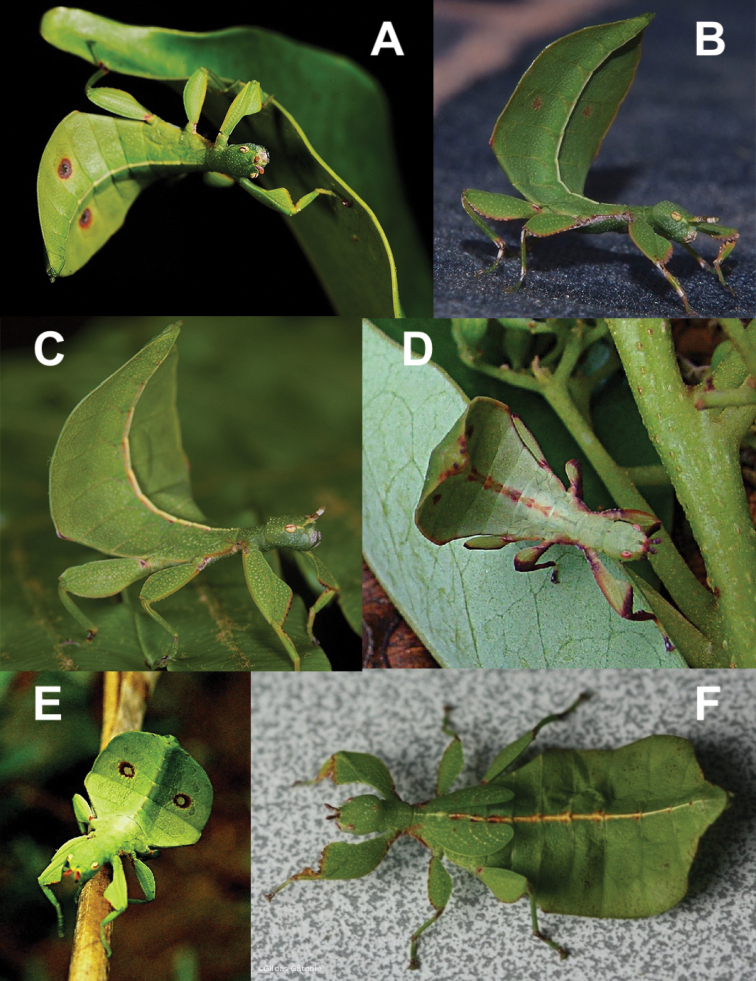
Live nymphs of *Trolicaphyllium* gen. nov. which could not be identified to species. Images from https://endemia.nc and used under creative commons license (CC BY-NC-SA 4.0) **A** Frédéric Desmoulins, Plaine des Lacs, April 2014 **B** Hendrik Oesterlin, Koé (Dumbéa) elevation 185 meters, June 2006 **C** Julien Barrault, Hienghène, November 2010 **D** Daniel and Irène Létocart, Tchamba, October 2009 **E** Bernard Suprin, Dumbéa, April 2004 **F** Gildas Gâteblé, Ouenghi, October 2012.

With significant confusion surrounding the distribution of *Trolicaphyllium* gen. nov. species in New Caledonia (due to apparent intraspecific variation and sexual dimorphism), this leaves many records as unidentifiable to species (see Suppl. material 1). Additionally, many records we have seen are of nymphs, which are rather difficult to estimate final adult morphology from (Fig. 30). These many records are still rather interesting as they can help to clarify the distribution of this New Caledonian endemic genus (Fig. 21). For example, a singular record of a male from the AMNH which was collected by Lindsay Macmillan on Maré Island during the Whitney South Sea Expedition and recorded as a “mantis” within the expedition journal, was located within the unidentified Mantodea drawer within the AMNH collection by the first author and represents one of only two records we have seen to date from this island (Macmillan 1938).

**Figure 31.**
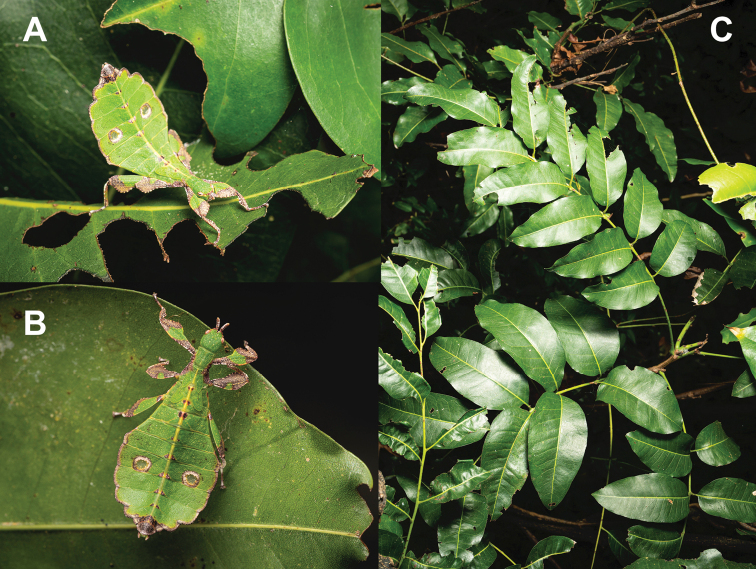
Unidentifiable *Trolicaphyllium* gen. nov. male nymph photographed on Tiga Island in August 2020 by Damien Brouste (New Caledonia) (iNaturalist user: damienbr) **A** dorso-anterior view **B** same nymph, dorsal habitus **C** unidentified host plant on which the nymph was recorded.

With phylliids typically rather island endemic, it is uncertain at this point if the records from Tiga (Fig. 31) and Maré islands are a currently described species (representing a range expansion), or if they are yet to be described species. The latter is possible given that no species have yet been described from these islands. Unfortunately, neither of these records could be sequenced as one was observational and the other too old to sequence, therefore little can be gleaned at this time regarding these islands. With *Trolicaphyllium* gen. nov. records known from Grande Terre, Lifou, Tiga, Maré, Ile de Bélep, and L’Île-des-Pins islands, we expect that possibly other small islands in New Caledonia such as Ouvéa may also have populations, but to date we are not aware of any observational records or specimens from additional islands (Fig. 21). Hopefully, future sequencing of museum specimens and additional local efforts to sample these smaller islands such as Lifou (which will be necessary as it is the type locality for *Trolicaphylliumbrachysoma* comb. nov.) will reveal the true distribution of *Trolicaphyllium* gen. nov. species in New Caledonia. Of particular use will likely be immature nymph specimens present within many museum collections which although not very useful for morphology, significantly increase the molecular data if sequenced and can add clarity to species boundaries/intraspecific sequence variation.

Only now are the higher-level relationships within the phylliids being explored, with the most likely sister group to *Trolicaphyllium* gen. nov. appearing to be the *Comptaphyllium* (Bank et al. 2021). Geographically speaking, one possibility for “filling in this missing gap” between the New Caledonian clade and the *Comptaphyllium* could be *Pulchriphylliumgroesseri* (Zompro, 1998), a morphologically unique species known only from the Solomon Islands between New Guinea and New Caledonia. This species is only tentatively placed within the *Pulchriphyllium* due to the presence of exterior lobes on the tibiae and similar tegmina venation, but due to the lack of fresh material to sequence, this species has not yet been included within any molecular phylogenies. Due to the similar body size and abdominal shape to certain *Trolicaphyllium* gen. nov. species we wonder if *Pulchriphylliumgroesseri* may indeed be related and could geographically link these two genera, therefore giving more clarity to the relationships within this group.

Additionally, geographically, it is possible that Vanuatu to the north of New Caledonia also has suitability for phylliids and may have been part of their route of colonization to modern day New Caledonia assuming an origin of New Guinea for modern phylliids (Bank et al. 2021). Interestingly however, to date Vanuatu lacks phylliid records which could be used as reference and no museum specimens have been located.

Morphologically it is not uncommon within the phylliids for single species to be rather variable within their abdominal shape, ranging from tapered, smooth and boxy, weakly lobed, to strongly lobed (Hennemann et al. 2009; Cumming et al. 2020b). Review of every museum specimen we could locate has shown a significant variability within the abdominal shape of many nymphs and multiple adults. We hope that future molecular reviews of *Trolicaphyllium* gen. nov. specimens of variable morphology from throughout Grande Terre and the surrounding islands will reveal how many species there likely are in New Caledonia. Within phylliids there appears to be vastly differing limits geographically for how far a single species will range, with numerous examples of island endemics (e.g., Cumming et al. 2018, 2019a, 2020a) as well as instances of single species spanning extensive geographic ranges (e.g., Cumming et al. 2020a, 2021). It is possible with *Trolicaphylliumbrachysoma* comb. nov. known from Lifou, and the others from Grande Terre, that there are at least two species (at least one from each island) and we hope that future molecular review of a wide sampling of specimens will reveal species geographic boundaries with more clarity. If molecular reviews of numerous specimens from Grande Terre reveal only a single morphologically variable species on Grande Terre, this would likely mean that only two species as presently known would be warranted *Trolicaphylliumbrachysoma* comb. nov. from Lifou and *Trolicaphylliumerosus* comb. nov. from Grande Terre, with *Trolicaphylliumsarrameaense* comb. nov. a junior synonym of *Trolicaphylliumerosus* comb. nov. Hopefully, future molecular reviews will help clarify the *Trolicaphyllium* gen. nov. diversity within New Caledonia.

### Functionally relevant morphological characters

Some morphological features separating *Chitoniscus* sensu stricto and *Trolicaphyllium* gen. nov., namely tarsal and egg morphology, can shed further light on the evolutionary history of these groups, as the morphological difference can be a result of adaptations that accompany the process of speciation. Their functional relevance can highlight the functional constraints which led to the specific morphological traits. The overall tarsus morphology in phasmids in general and in Phylliidae in particular is quite conserved, but the differences found are probably results of adaptations to the specific environments of the species (Büscher and Gorb 2017; Büscher et al. 2018a, b). While the male tarsus of both groups is similar and not different in morphology when compared to other Phylliidae (Büscher et al. 2019), the female tarsi differ in one functional feature. The tarsi of *Trolicaphylliumsarrameaense* com. nov. females bear an extension in form of a median ridge of the euplantulae on the tarsomeres 2–4 (Fig. 12B), which is so far not reported for any other phasmid in this form. This accessory ridge can elongate the adhesive surface of the tarsus distally and increase traction along the length of the tarsus, as euplantulae are primarily used for generation of friction in phasmids (Busshardt et al. 2012; Labonte and Federle 2013; Büscher and Gorb 2019; Büscher et al. 2020a). However, the arrangement of the euplantulae stabilizes the attachment primarily in the proximal-distal direction, which could be beneficial in the typical postures of these insects in which their legs are arranged circular around the stout body. Furthermore, the elongation of the tarsal chain increases traction on thin stems, if the tarsi grasp around the substrate (Büscher et al. 2020a). A similar effect is additionally achieved by the accessory euplantula on tarsomere 5. This feature is consistently present in all investigated Phylliidae but lacking in many other phasmid species (Vallotto et al. 2016; Büscher et al. 2019).

The eggs of both groups differ significantly in the modification of their exochorionic surface. While *Trolicaphyllium* gen. nov. bear distinct microscopic spherical structures on the surface of the eggs (Fig. 17A–D), the eggs of *Chitoniscus* sensu stricto have a rough, porous surface. These surfaces can be fundamentally different in their function. The spherical structures might be water and dirt repellent, while the rough, porous surface can facilitate water spreading or uptake (Watson et al. 2017). These functions, however, need to be tested in subsequent studies to corroborate their potential benefits.

The presence or absence of pinnae and their respective morphology have been shown to be of taxonomic value for the eggs of Phylliidae already (Clark 1978; Cumming et al. 2020a), this feature, however, is also of striking functional relevance (see. Büscher et al. 2020b, c). The exochorionic pinnae of the eggs of *Phylliumphilippinicum* are involved in a water responding adhesive system that attaches the eggs to different surfaces (Büscher et al. 2020b). Similar fringe-like expansions on their egg shells are reported for other phylliid eggs and hypothesized to be involved in adhesion as well (Büscher et al. 2020c). Due to their similarity in morphology, it is highly likely that the eggs of *Chitoniscus* sensu stricto provide the same adhesive capability. These structures probably also carry a glue and expand when they come in contact with water and distribute glue on the corresponding surface. Whether the mushroom-like elements on the surface of *Trolicaphyllium* gen. nov. eggs provide adhesion and how their potential adhesion responds to water is so far unknown and could be tested in subsequent studies. This could also yield a more elaborate analysis of the evolution of adhesion in the eggs of Phylliidae including closely related lineages with different pinna morphologies.

To conclude, despite more than a century of considering the Fijian and New Caledonian phylliids a single genus, we herein adjust the taxonomy of this polyphyletic clade. Our erection of the *Trolicaphyllium* gen. nov. reflects the unique aspects of the species of New Caledonia as distinct from their previously considered congenerics (*Chitoniscus* sensu lato).

Herein we have addressed the several questions presented in the introduction regarding these two groups of leaf insects. Firstly, although these clades are in general similar due to their smaller size and abdominal shapes ranging from smoothly tapered to boxy and strongly lobed, a thorough review of small and microstructures of adults, nymphs, and eggs present a plethora of morphological features for easy and reliable differentiation (Table 1). Secondly, although molecular data for species from these two countries is rather limited, all results have been consistent and in agreement with our morphological findings, with the two clades always corresponding to the geographic distributions (Fiji always sister to the greater leaf insects and New Caledonia a distinct clade nested within the greater phylliids). Thirdly, based upon our review, we assessed what taxonomic option would most clearly correct this issue. With such drastically different morphologies for these two clades, as well as their unique morphologies from all other extant phylliids, our most transpicuous taxonomic path was for clarifying these as genera.

Therefore, based upon years of molecular results, dozens of morphological features, and the significant geographic isolation for these two clades of leaf insect, we feel that the erection of *Trolicaphyllium* gen. nov. properly addresses the many discrepancies which were glaringly problematic with the *Chitoniscus* sensu lato.

## Supplementary Material

XML Treatment for Trolicaphyllium

XML Treatment for Trolicaphyllium brachysoma

XML Treatment for Trolicaphyllium erosus

XML Treatment for Trolicaphyllium sarrameaense

## Supplementary materials

Supplementary material 1 Table S1. Material examined (specimen data and deposition/ observational records) for localities used within the distribution map (Fig. 21) and to accompany the discussion of each species Data type specimen data https://binary.pensoft.net/file/574826This dataset is made available under the Open Database License (http://opendatacommons.org/licenses/odbl/1.0/). The Open Database License (ODbL) is a license agreement intended to allow users to freely share, modify, and use this Dataset while maintaining this same freedom for others, provided that the original source and author(s) are credited.Royce Cumming, Stéphane Le Tirant, Thies H. Büscher
